# Geographical and Historical Patterns in the Emergences of Novel Highly Pathogenic Avian Influenza (HPAI) H5 and H7 Viruses in Poultry

**DOI:** 10.3389/fvets.2018.00084

**Published:** 2018-06-05

**Authors:** Madhur S. Dhingra, Jean Artois, Simon Dellicour, Philippe Lemey, Gwenaelle Dauphin, Sophie Von Dobschuetz, Thomas P. Van Boeckel, David M. Castellan, Subhash Morzaria, Marius Gilbert

**Affiliations:** ^1^Spatial Epidemiology Lab (SpELL), Université Libre de Bruxelles, Brussels, Belgium; ^2^Food and Agriculture Organization of the United Nations, Rome, Italy; ^3^Department of Microbiology and Immunology, Rega Institute, KU Leuven — University of Leuven, Leuven, Belgium; ^4^Institute of Integrative Biology, ETH Zurich, Zurich, Switzerland; ^5^Center for Disease Dynamics, Economics and Policy, Washington, DC, United States; ^6^DM Castellan Consulting, Niagara Falls, ON, Canada; ^7^Fonds National de la Recherche Scientifique (FNRS), Brussels, Belgium

**Keywords:** novel HPAI emergences, low pathogenic to highly pathogenic conversion, reassortment, spatial and temporal pattern, phylogeography

## Abstract

Over the years, the emergence of novel H5 and H7 highly pathogenic avian influenza viruses (HPAI) has been taking place through two main mechanisms: first, the conversion of a low pathogenic into a highly pathogenic virus, and second, the reassortment between different genetic segments of low and highly pathogenic viruses already in circulation. We investigated and summarized the literature on emerging HPAI H5 and H7 viruses with the aim of building a spatio-temporal database of all these recorded conversions and reassortments events. We subsequently mapped the spatio-temporal distribution of known emergence events, as well as the species and production systems that they were associated with, the aim being to establish their main characteristics. From 1959 onwards, we identified a total of 39 independent H7 and H5 LPAI to HPAI conversion events. All but two of these events were reported in commercial poultry production systems, and a majority of these events took place in high-income countries. In contrast, a total of 127 reassortments have been reported from 1983 to 2015, which predominantly took place in countries with poultry production systems transitioning from backyard to intensive production systems. Those systems are characterized by several co-circulating viruses, multiple host species, regular contact points in live bird markets, limited biosecurity within value chains, and frequent vaccination campaigns that impose selection pressures for emergence of novel reassortants. We conclude that novel HPAI emergences by these two mechanisms occur in different ecological niches, with different viral, environmental and host associated factors, which has implications in early detection and management and mitigation of the risk of emergence of novel HPAI viruses.

## Introduction

Highly Pathogenic Avian Influenza (HPAI) viruses of the H5 and H7 subtypes represent a global human health concern (1) in addition to causing severe economic losses in the poultry industry (2). These viruses have an eight-segmented genome, and undergo frequent genetic reassortment and mutations leading to creation of genetic diversity and emergence of novel viruses.

The natural reservoir of avian influenza (AI) diversity is the wild bird ecosystem, where all subtypes circulate in the low pathogenic (LP) form with in various combinations of one of the 16 haemaggultinin (H1–H16) and one of the nine neuraminidase (N1–N9) surface protein genes (3). Frequent transmission of different AI viruses and their genetic segments between wild bird host species, especially in the orders Anseriforms and Charadriiformes favours maintenance of avian influenza genetic diversity. In addition, contact rates can increase during migration periods, such as in stopover sites, where the host diversity is significant with several species, age groups, of different immune status congregating together. This considerable diversity in host range selects sets of virus subtypes in the low pathogenic form that are capable of maintaining transmission cycles through different hosts (4). In this system, there is predominantly environmental transmission between hosts, with evidence of lower evolutionary rates maintaining evolutionary stasis (5). Thus, virus evolution is characterized by low virulence, and high host survival rates; two conditions that are compatible with long distance migration. In summary, epizootics caused by HPAI in wild bird populations are seldom, and were mostly documented for virus strains that had previously been associated with poultry farming (6). 

Poultry farms and their associated value-chain networks form the secondary system for AI transmission. On a host level, these poultry systems are characterized by single or limited host species (primarily galliform poultry and waterfowl), of uniform age and considerably lower genetic diversity, reared in high-density flocks, though those factors vary greatly according to the intensification level of the poultry production system considered (7). In high-income countries (HICs), over 95% of chickens are raised intensively in commercial and highly specialized poultry production systems for eggs and meat production (8). In contrast, in low-income countries (LICs), the large majority of poultry is still raised in backyard extensive poultry production systems, for subsistence and as a way to generate income in rural settings. A full range of intermediate situations exists in between these two extremes, which follow a gradient of income. Middle-income countries (MICs) typically face an intermediary situation where both extensive and intensive poultry production systems co-exist (8–10), and where value-chains involve a number of intermediate workers and live bird markets. These poultry agro-ecosystems can be broadly divided between regions that are mostly free from HPAI and where HPAI outbreaks are sporadic, detected and contained early; and regions where HPAI is endemic or showing frequent reoccurrences, and where there are challenges associated with detection and response. The poultry agro-ecosystem is characterized by anthropogenic transmission risks linked to the farming system and value-chains, a lower diversity of circulating types and sub-types, spillover between host species and occasional emergence of novel HPAI viruses.

The modes of evolution of AI viruses within these two systems vary according to the evolutionary pressures accompanying each system. In wild birds, novel AI viruses evolve using two main mechanisms. First, continuous accumulation of point mutations, deletions and substitutions due to lack of proof reading in the RNA polymerase creates antigenic drift (11). Second, exchange of genetic segments between two co-infecting viruses within a host cell leads to genetic reassortment and yields novel subtypes by antigenic shift. These modes of evolution predominantly result in LPAIV subtypes causing subclinical infections (12). An additional fact is that in countries where long term surveillance is conducted in the wild bird system (over 43 years), no novel emerging HPAIV subtypes have ever been isolated (13).

Within the poultry systems, genetic mutations are constantly occurring, and the emergence of novel HPAIVs have been reported on a regular basis in relation to the following main mechanisms. The presence of a multibasic cleavage site (MBCS) within the HA is one of the properties used by World Organization for Animal Health (OIE) to classify AI viruses as highly pathogenic (14). There are several mechanisms by which this can occur; first, new HPAIVs can emerge from the acquisition of certain stochastic mutations of nucleotide/amino acid substitution leading to the insertion of basic amino acids yielding (MBCS) in the HA gene of an existing LPAI virus (15, 16). The MBCS can also be introduced into the HA gene in an LPAI by recombination with host or viral RNA (17), which only occurred on a few occasions in the H7 subtype (18). Third, a novel HPAIV can emerge from the reassortment between already circulating LPAIV and HPAIV influenza viruses by exchange of genetic segments (12).

In this paper, we aim to describe the conditions of emergence of novel HPAIV in the poultry system. Two main methods of novel HPAIV emergence will be reviewed: the acquisition of the MBCS in an LPAI virusus leading to conversion to HPAI- called as “conversions”, and the exchange of genetic segments between viruses leading to generation of a novel HPAI, called “reassortments”. A data set of these conversion and reassortment events will be compiled in order to describe their geographical distribution and spatio-temporal trends in relation to poultry production systems. Finally, we will use phylogeography to assess the evolutionary and geographical relationships between these novel HPAI emergences.

## Methods

### Compilation of Conversion and Reassortment Datasets:

### Case Definitions

*HPAI Conversions*: The first reports of novel H5 or H7 HPAI viruses which are documented as resulting from the conversion of a LP to a HP strain subsequent to introduction from wild birds and circulation and gain of pathogenicity in poultry were classified as HPAI Conversions.The gain in pathogenicity should have resulted from the insertion of a MBCS in the HA of a LP virus. Only the primary/first report of emergence of an HPAI in poultry was considered, and the secondary spread of the same subtype during an epidemic was excluded. The dataset of HPAI conversions was compiled from 1959 onwards.

*HPAI Reassortments:* The first reports of novel HPAI viruses generated by inter and intra subtype exchanges of genetic segments were classified as “HPAI-reassortants”. Reports HPAI H5 and H7 novel reassortment events were compiled using methods described below. Novel reassortant viruses isolated from primary outbreaks, surveillance of live bird markets (LBMs), wild-bird die-off events, and from human and other mammalian cases of HPAI caused by H5 and H7 subtypes were included in the dataset. The dataset of HPAI reassortments was compiled from 1996 onwards, as we could not find any reports of reassortments prior to that.

It is to be noted, that the primary report of a novel HPAI emergence may not actually be entirely accurate as the primary report actually pertains to a first detection/isolation, which may reflect a surveillance bias and indeed may not be the actual conversion/reassortment event.

### Methodology

Internet searches using Google Scholar and PubMed databases for conversions were performed using keywords, “H5/H7 LPAI to HPAI”, “LPAI to HPAI outbreaks”, “ H5/H7 LPAI to HPAI Conversion ”, and “H5/H7 LP to HP emergence”. For reassortments, the keywords used were “Novel Reassortment”, “HPAI Reassortment”, “HPAI AND novel reassortant”, “Novel HPAI”, “Novel emergence” along with H5/H7 keywords. We also looked at the paper cited, or being cited by the papers found using the search terms in order to find further references that would not have been found in the primary search.

Thereafter, internet search engines (Internet explorer/Google) were also searched used using similar keywords. Time filters were applied on these searches by dividing into three time periods: up to 1995, 1996–2005, and 2006–2015. Country names were also added to these keywords to further find epidemiological details regarding these events. The publications citing these novel emergences were reviewed to classify the report into a conversion or reassortment event based on the documented evidence. In several instances, the references within a publication would reference other novel emergences, and these references were also reviewed for addition of events to the dataset.

The geographic coordinates of the outbreak/isolation were recorded. For some of the earlier events, the geographic coordinates are not exact, and are available only at coarse resolutions. For the reassortments especially with reference to China and Vietnam, several isolates were from LBM surveillance and the exact location could not be ascertained beyond administrative level 2 or 3. The time of outbreak/isolation was also obtained, and for the purpose of homogeneity, we kept the time period, as “year” as exact dates of sampling were not available for most viruses. The subtype (HxNx) involved in the conversion and reassortment event was recorded along with the host species and type of poultry farming system (backyard poultry, commercial poultry and wild birds) of the first isolation of the novel HPAI subtype. Where necessary, the information on outbreaks/isolates was crosschecked in publicly available databases, which included the EMPRES-i database (http://empres-i.fao.org/eipws3g/), the OIE WAHID database (14), and the ProMed-mail (www.promedmail.org) to verify the host species, production system, geographic location and add any other information that may be available.

The sequence isolated from each conversion and reassortment event was identified from the literature along with its accession number. The isolate name and accession numbers of the submitted sequences were obtained from GenBank^®^ (www.ncbi.nlm.nih.gov/genbank/) (19), the Global Initiative on Sharing All Influenza Data (GISAID) EpiFlu Database (http://www.gisaid.org) and the Influenza Research Database (http://www.fludb.org/brc/search_landing.spg?decorator=influenza). In addition, source details of the associated host species, subtype/gene segment involved in the generation of the reassortant isolate were also tabulated as described by the referenced publication. The accession numbers of four sequences for conversion events could not be found in either of the databases, as the respective authors may not have submitted them yet. We were able to obtain the accession number of all reassortant isolates from the public databases. The publication citing the conversion and reassortment was also referenced for each novel virus. In some cases multiple strains were cited in the publication, but only some could be classified as a novel reassortants, therefore careful scrutiny of each publication was carried out to include only new reassortants.

### Analysis

The data sets were mapped according to three study periods: Up to 1995, 1996–2005, and 2006–2015. These were chosen as they historically represented times at which important changes occurred in the epidemiology of HPAI: the year 1996 marked the emergence of the HPAI H5N1 A/Goose/Guangdong/1/96 virus, which was distinct from the earlier sporadically occurring HPAI H5 subtypes. During this period, geographic spread within southeast and east Asia of the Gs/Gd lineage was documented. Period three was chosen as the year 2006 marked the period of global expansion of HPAI from Asia into the Indian subcontinent, Europe and Africa.

In order to estimate the evolutionary relationships between the selected H5 and H7 sequences associated with conversions and reassortment events, we used the discrete phylogeographic approach implemented in BEAST 1.8.4 (20). This Bayesian phylogenetic diffusion model allows inferrering ancestral times and spatial locations at the internal and root nodes of time-calibrated phylogenetic trees (21). A distinct analysis was performed for the H5 and H7 alignments, for which we partitioned the coding genes into first + second and third codon positions. In BEAST, we specified a general GTR+Γ + I nucleotide substitution model, an uncorrelated lognormal relaxed molecular clock to account for evolutionary rate variation among lineages (22) and a flexible Bayesian Skyride coalescent tree prior (23). The discrete diffusion model was parametrized as a nonreversible continuous-time Markov chain (CTMC) model (24). We used the program Tracer 1.6 (20) to ensure that effective sample sizes were greater than 200 for all the different parameters estimated by BEAST. Finally, TreeAnnotator 1.8.4 (20) was used to summarize the inferred ancestral locations from the resulting posterior distribution on a maximum clade credibility (MCC) tree.

## Results

**Figure 1:**
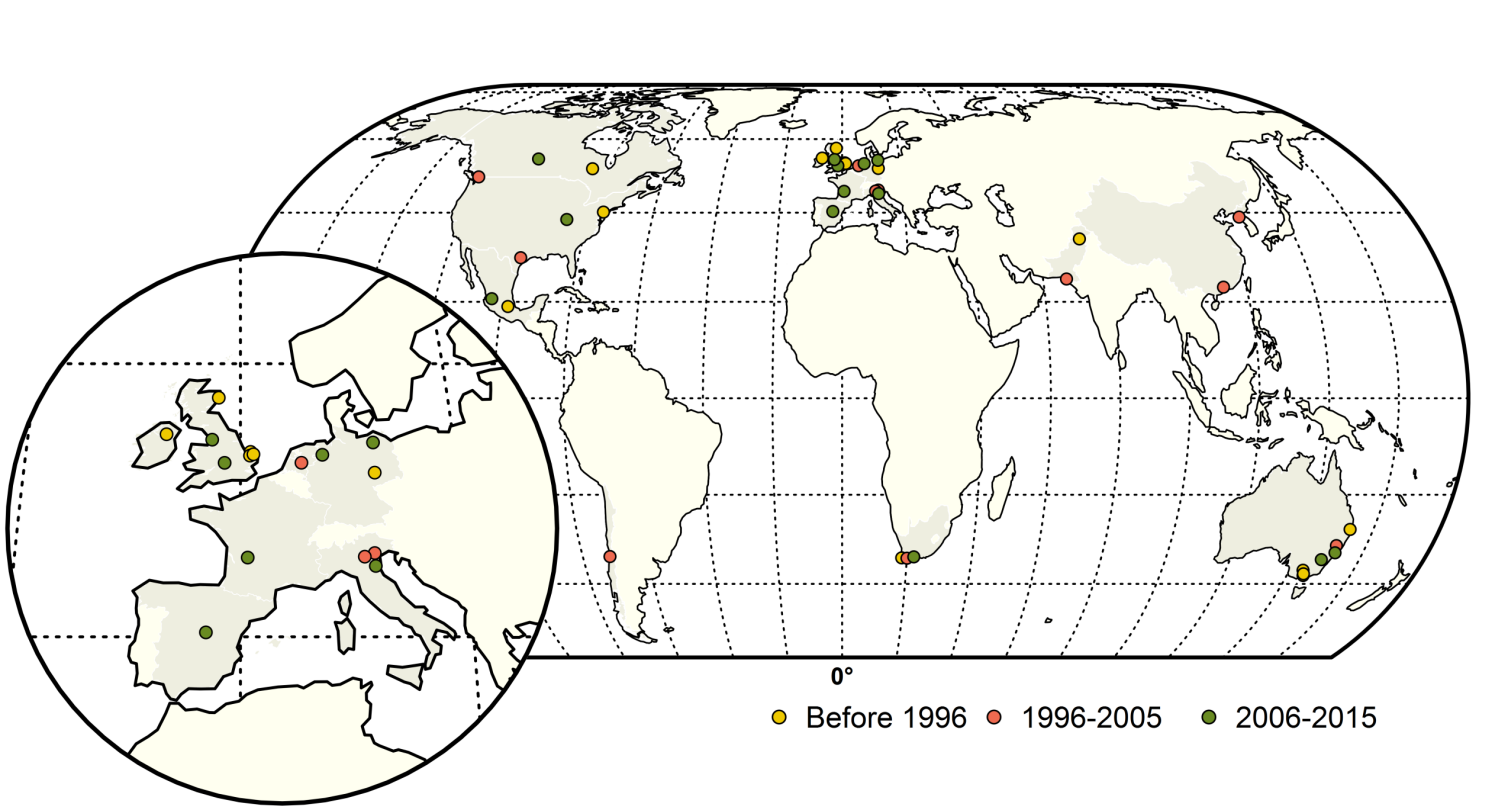
Map of highly pathogenic avian influenza (HPAI) subtype H5 and H7 conversions during three time periods; yellow (before 1996), red (1996–2005) and green (2006–2015). The shapefile data used to produce these maps were made with Natural Earth (http://www.naturalearthdata.com/). The graticule is composed of a 20-degree increments and the coordinate system is Eckert IV (EPSG: 54012).

**Figure 2:**
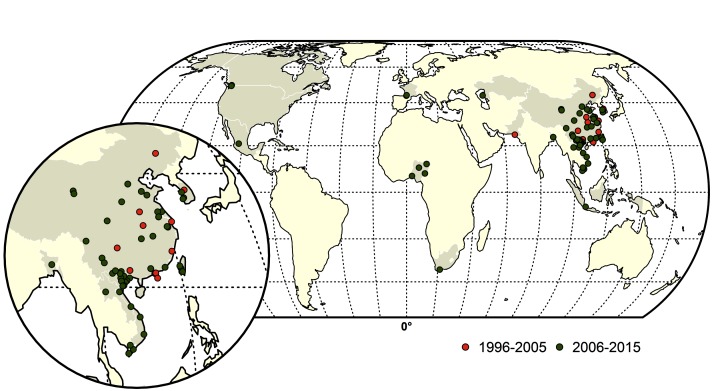
Map of Highly pathogenic avian influenza (HPAI) subtype H5 and H7 reassortments during two time periods; red (1996–2005) and green (2006–2015). The shapefile data used to produce these maps were made with Natural Earth (http://www.naturalearthdata.com/). The graticule is composed of a 20-degree increments and the coordinate system is Eckert IV (EPSG: 54012).

From 1959 to 2015, a total of thirty-nine LPAI to HPAI H5 (*n* = 14) and H7 (*n* = 25) conversion events were documented (Figure 1). A total of 127 HPAI reassortments were documented since 1959 (Figure 2; Table S1 in SI) and only two of the reassortments were reported from H7 subtype, while the remainder was all reported in H5 subtype. Spatially, a large majority of the conversions occurred in Europe (*n* = 14), followed by the Americas (*n* = 9), Australia (*n* = 7), Africa (*n* = 3) and Asia (*n* = 4). The details of all the conversion events are provided in Table 1. In contrast, the reassortments were concentrated in Asia (117), with only a few in Africa (*n* = 5), Americas (*n* = 3) and Europe (*n* = 2). There were no reassortments reported from Australia. Within Asia, the highest number of reassortments were documented in China (*n* = 56), followed by Vietnam (*n* = 35). Hong Kong (*n* = 11) Taiwan (*n* = 4), Korea and Bangladesh (*n* = 3), Indonesia (*n* = 2), DPR Korea and Kazakhstan reporting only one reassortment. In Africa, four reassortment events were reported from Nigeria and one from South Africa. Europe reported two reassortment events. In the Americas, Mexico, Canada and the USA reported one HPAI reassortment each. The details of all the HPAI reassortments events are provided in Table S1 in SI.

**Table 1:** List of Highly Pathogenic Avian Influenza Conversion (subtype H5 and H7).

**Country**	**Year**	**Subtype**	**Location**	**Species**	**Wildbird Link**	**Interface**	**AccessionNo**	**Reference**	**Strain Name Strain No.**
***Time Period: before 1996***									
UK	1959	H5N1	Scotland	Commercial, Chickens	Y	Interface	X07826	(25)	(A/chicken/Scotland/1959(H5N1)
South Africa	1961	H5N3	Capetown	Wild terns	Y	Interface	U20460	(26)	(A/tern/South Africa/1961(H5N3)
UK	1963	H7N3	England	Commercial, Turkeys	Y	Interface	U20462	(27)	(A/turkey/England/1963(H7N3)
Canada	1966	H5N9	Ontario	Commercial, Turkeys	N	Poultry	CY107859	(28)	(A/turkey/Ontario/7732/1966(H5N9)
Australia	1976	H7N7	Victoria	Commercial, Chickens	N	Poultry	CY061602	(29)	(A/duck/Victoria/1976(H7N7)
UK	1979	H7N7	England	Commercial, Turkeys	Y	Interface	M30122	(30)	A/turkey/England/199/79 (H7N7)
Germany	1979	H7N7	Leipzig	Commercial, Chickens	Y	Interface	U20459	(31)	A/chicken/Leipzig/79 (H7N7)
USA	1983	H5N2	Pennsylvania	Commercial, Chickens	N	Poultry	CY107848	(32)	(A/chicken/Pennsylvania/1370/1983(H5N2)
Ireland	1983	H5N8	Ireland	Commercial, Turkeys	Y	Interface	CY015089	(33.	(A/turkey/Ireland/1378/1983(H5N8)
Australia	1985	H7N7	Victoria	Commercial, Chickens	Y	Interface	CY025069	(34)	(A/chicken/Victoria/1/1985(H7N7)
UK	1991	H5N1	East Anglia	Commercial, Turkeys	Y	Poultry	EU636692	(35)	(A/turkey/England/50-92/91(H5N1)
Australia	1992	H7N3	Victoria	Commercial, Chicken, Ducks	N	Poultry	CY025077	(36)	(A/chicken/Victoria/224/1992(H7N3)
Australia	1994	H7N3	Queensland	Commercial, Chickens	Y	Interface	CY022685	Rev. sci. tech. Off. int. (37)	(A/chicken/Queensland/1994 (H7N3)
Mexico	1994	H5N2	Puebla state	Commercial, Chickens	N	Poultry	L46586	(15)	(A/Chicken/Puebla/8623-607/94(H5N2)
Pakistan	1994	H7N3	Islamabad	Commercial, Chickens	N	Poultry	AF202226	Naeem and Hussein. 1995	A/chicken/Pakistan/447/94 (H7N3)
***Time Period: 1996–2005***									
**Country**	**Year**	**Subtype**	**Location**	**Species**	**Wildbird Link**	**Interface**	**AccessionNo**	**Reference**	**Strain Name Strain No.**
China	1996	H5N1	Guangdong Sheng	Commercial, Geese	Y	Interface	AF144305	(38)	(A/Goose/Guangdong/1/96(H5N1)
Australia	1997	H7N4	New South Wales	Commercial, Chickens	Y	Interface	CY022701	(39)	(A/chicken/New South Wales/327/1997 (H7N4)
Italy	1997	H5N2	Veneto and Friuli-Venezia Giulia regions	Backyard, Chicken	Y	Interface	GU052403	(40)	(A/chicken/Italy/330/97 (H5N2)
Italy	1999	H7N1	Verona	Commercial, Turkeys	N	Poultry	DQ991320	(41)	A/Chicken/Italy/5093/1999 (H7N1)
Chile	2002	H7N3	San Antonio, V Region	Commercial, Chickens	N	Poultry	AY303632	(17)	(A/chicken/Chile/4957/02(H7N3))
Pakistan	2003	H7N3	H7N3	Commercial, Chickens	N	Poultry	FJ577526	(42)	(A/chicken/Karachi/NARC-23/2003(H7N3)
Netherland	2003	H7N7	Gelderland	Commercial, Chickens	Y	Poultry	AY338458	(43)	(A/chicken/Netherlands/1/03(H7N7)
Canada	2004	H7N3	Fraser Valley, British Columbia	Commercial, Chickens	N	Poultry	AY731820	(44)	(A/chicken/British Columbia/NS-1390-2/04(H7N3)
USA	2004	H5N2	Texas	Commercial, Chickens	N	Poultry	AY849793	(45)	(A/chicken/TX/298313/04(H5N2))
South Africa	2004	H5N2	Western Cape	Commercial, Ostrich	Y	Interface	FJ519983	(46)	(A/ostrich/South Africa/N227/2004(H5N2))
Dem People's Rep of Korea	2005	H7N7	P'yongyang-si	Commercial, Chickens	N	Poultry		ftp://ftp.fao.org/docrep/fao/010/a0115e/A0115E04.pdf	
***Time period: 2006–2015***									
**Country**	**Year**	**Subtype**	**Location**	**Species**	**Wildbird Link**	**Interface**	**AccessionNo**	**Reference**	**Strain Name Strain No.**
Canada	2007	H7N3	Saskatchewan	Commercial, Chickens	Y	Interface	EU500860	(47)	(A/chicken/SK/ HR-00011/2007 (H7N3))
UK	2008	H7N7	England	Commercial, Chickens	N	Poultry	FJ476173	(48)	(A/chicken/England/1158-11406/2008(H7N7))
Spain	2009	H7N7	Castilla-la Mancha	Commercial, Chickens	Y	Interface	GU121458	(49)	(A/chicken/Spain/6279-2/2009(H7N7))
South Africa	2011	H5N2	Western Cape	Commercial, Ostrich	Y	Interface	JX069081	(50)	(A/ostrich/South Africa/AI2114/2011(H5N2))
Mexico	2012	H7N3	Jalisco	Commercial, Chickens	Y	Poultry	JX397993.1	(51)	(A/chicken/Jalisco/12283/2012)(H7N3)
Australia	2012	H7N7	New South Wales	Commercial, Chickens	Y	Interface		FAO Empres-I	NA
Italy	2013	H7N7	Po river delta of Emilia Romagna Region	Commercial, Chickens	Y	Interface	KF569186	(52)	(A/chicken/Italy/13VIR4527-11/2013(H7N7))
Australia	2013	H7N2	New South wales	Commercial, Chickens	Y	Interface	NA	http://www.dpi.nsw.gov.au/__data/assets/pdf_file/0003/458553/AHS-oct-dec-2012.pdf.	NA
Germany	2014	H5N8	MecklenburgVorpommern	Commercial, Turkeys	Y	Interface	EPI544756	(53)	(A/turkey/Germany-MV/AR2472/2014; AR2472/14)
Germany	2015	H7N7	NIEDERSACHSEN	Commercial, Chickens	Y	Interface		(54)	NA
UK	2015	H7N7	Lancashire	Commercial, Chickens	Y	Interface	EPI623939	(54)	A/chicken/England/26352/2015 (H7N7)
France	2015	H5N1	Dordogne	Backyard, Chicken	Y	Interface	KU310447	(55)	(A/chicken/France/150169a/2015(H5N1)
USA	2015	H7N8	Indiana	Commercial, Turkeys	N	Poultry	KU558906	(56)	A/turkey/Indiana/16-001403-1/2016(H7N8)

Temporally, the conversion events were reported throughout the three time periods, with 15, 11 and 13 events in the 1959–1995, 1996–2006 and 2006–2015 periods, respectively. In contrast, up to the year 1995, no HPAI reassortments were reported in the literature. In the following decade, 45, and in the period of 2006–2015, a total of 82 reassortments were documented respectively. The spatio-temporal pattern of reassortment reports largely followed the spread of the HPAI H5N1 virus, which reassorted with many other viruses giving rise to several H5Nx viruses, as detailed in the Supplementary Information Data Sheet S1.

From 1959 to 1995, the conversion events were spatially dominant in Europe and Australia. The majority of conversions occurred in the UK (1959 H5N1, 1963 H7N3, 1979 H7N7, 1991 H5N1) and Ireland (1983 H5N8) with both the H7 and the H5 subtypes involved. The continent of Australia was affected four times with only H7 LP to HP conversions during this period (1976 and 1985 H7N7, 1992 and 1994 H7N3). Single conversion events occurred in USA (1983 H5N2), Canada (1966 H5N9), South Africa (1961 H5N3), Germany (1979 H7N7), Mexico (1994 H5N2) and Pakistan (1994 H7N3).

During the next decade from 1996 to 2005, Europe still remained the center of conversions, however, unlike the previous time period, none of the events were recorded in the UK. Instead Italy (1997 H5N2, 1999 H7N1), and the Netherlands (2003 H7N7) were affected by outbreaks caused by LP to HP conversions. Interestingly Australia had only one conversion (1997 H7N4) event. Canada (2004 H7N3) and the USA (2004 H5N2) had one event each, similar to the earlier time period. Conversions were also reported from South Africa (2004 H5N2) and Chile (2002 H7N3).

During this period, Asia reported conversions for the first time in Pakistan (2003 H7N3), DPR Korea (2005 H7N7) and China (1996 H5N1). The 1996 H5N1 virus became the progenitor of the HPAI H5N1 virus that began spreading across continents in the following decade.

From 2006–2015, there was a considerable increase in conversion events in Europe (especially towards the latter part of the decade) with seven conversions reported. Two conversions were reported from the Americas and Australia (2012 H7N7, 2013 H7N2). There were no conversions reported from Asia. During the initial part of the decade, the conversions were limited to the UK (2008 H7N7), Spain (2009 H7N7), Canada (2007 H7N3), South Africa (2011 H5N2), Mexico (2012 H7N3) and Italy (2013 H7N7). From 2014–2015 there was a quick succession of conversion events reported from Europe from Germany (2014 H5N8, 2015 H7N7), the UK (2015 H7N7) and France (2015 H5N1). Additionally, a LP to HP conversion involving H7N8 was reported in the USA in 2015. All conversion events are described in Table 1, and more details on these events can be found in Supplementary Information (SI) Data Sheet S2A.

In terms of host species and production systems, conversion events are predominant in intensive production systems with conversion events found in commercial chicken farms (*n* = 25), followed by commercial turkey farms (*n* = 8), ostrich farms (*n* = 2) and commercial geese farms (*n* = 1). Backyard farms only reported HPAI conversions on two instances: in 1997, in Italy with an HPAI H5N2 virus, and in France with an HPAI H5N1 virus. The only instance of a conversion being documented in wild birds was the large die-off of wild terns caused by HPAI H5N3 in 1961, off the coast of South Africa (26). Evidence of a direct interface with wild birds was reported in 19 of the 41 conversion events. This includes proximity to areas inhabited by wild birds, areas of overlap with migratory bird flyways and direct links established with wild bird sequences through phylogenetic analyses.

In comparison, the majority of the reassortants were isolated from chicken and ducks during live bird market (LBM) surveillance conducted in China and Hong Kong (*n* = 42). Other than that, 6 reassortants were isolated from geese samples and a single isolate came from a partridge sampled in LBMs. In Bangladesh, three reassortant isolates were isolated from chickens in LBM and poultry farms. In Vietnam, most reassortants were isolated from poultry farms from chickens (*n* = 15), ducks (*n* = 17), and a single isolate from ostrich and quail each from surveillance following outbreaks. Reassortant viruses were also isolated from commercial chicken farms in Mexico, Canada, China, Hong Kong, DPR Korea, Nigeria, Lao PDR and Taiwan. In Taiwan, three reassortants were also isolated from commercial goose farms. In France, two reassortant viruses were isolated from fattening duck farms.

Reassortants (*n* = 3) were also reported in commercial ostrich farms from South Africa, Nigeria and Vietnam. Six reassortants HPAI viruses were isolated from human cases, of which three were from China and one each from Hong Kong and Vietnam. Reassortant isolates were obtained from wild birds following reports of die-offs in 9 instances. Reassortants were also isolated from sparrows (*n* = 1), swine (*n* = 1), cats (*n* = 1) and a captive tiger (*n* = 1). To summarize, the large majority of the 127 HPAI H5 and H7 reassortants were reported from chicken (*n* = 51), ducks (*n* = 46), and geese (*n* = 8), and only few of them were sampled from other bird species.

**Figure 3:**
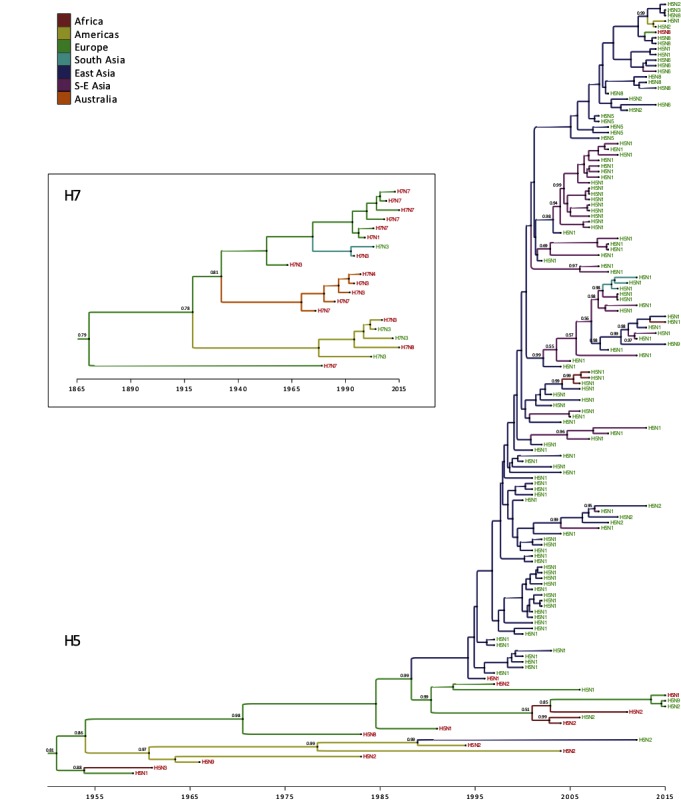
Time-scaled phylogeographic history of H5 and H7 sequences associated with HPAI conversions and reassortments. Branch colors represent the most probable location of the parental node of each branch. Tip labels indicate the subtype and are colored according to the associated event, i.e., conversion (in red) or reassortment (in green). When they are lower than 0.95, posterior probabilities of the most probable ancestral state (i.e. geographic location) are reported next to internal nodes.

We were able to obtain the accession numbers of all LPAI to HPAI conversions events, except four sequences (Table 1). For the reassortments, all accession numbers for the HA sequences could be obtained (Table S1 SI). The time scaled phylogeographic history of the available HA H5 and H7 subtype novel viruses produced by conversions and reassortments are presented in Figure 3, and present the time, region and subtype, that the conversion or reassortment event is associated with.

Within the H5 subtype, one can observe the dominance of conversion events until 1995, and these were largely restricted to Europe and America (Figure 3) apart from a single conversion of H5N3 into HPAI causing an outbreak in wild terns off the coast of South Africa. Sometime around late 1980s, the H5N2 subtype was introduced from the Americas (Mexico) into Taiwan (East Asia), which caused a reassortment between a Mexican-like H5N2 virus and a locally circulating H6N1 virus (57) and yielded a virus that was very similar to the vaccine strain used in Mexico. From 1995 onwards, with the emergence of the Gs/Gd HPAI H5N1 subtype, the emergence of novel reassortments started to be reported from eastern Asia (China and Hong Kong) and reassortments were only described as being of the H5N1 subtype until 2008–09. Introduction of HPAI of the H5 subtype to Southeast Asia took place around year 2000, which was followed by the regular isolation of multiple reassortant genotypes and subtypes. Multiple introductions followed by reassortments are also reported from East Asia (China) into Southeast Asia, indicating a continuous gene flow from eastern China into primarily Vietnam. The spread of H5 HA from China into Africa around 2005 is also noticeable, leading to the emergence and evolution of the European-Middle Eastern-African (EMA) (58) lineage that clusters in Africa. The HA of the South Asian H5N1 viruses also groups together (Figure 3, annotated in blue) indicating a single introduction from Southeast Asia followed by reassortments due to enzootic circulation of subtype H9N2. From 2009 onwards, the H5Nx reassortants start emerging, initially restricted to China, followed by expansion into Southeast Asia, and then global spread into Europe. Simultaneously, reassortment between the Asian H5N1 with the North American viruses yielded to the reassortant HPAI H5N1 and H5N2 in 2014 (Figure 3).

The H7 HA phylogeography does not provide any evidence for conversion or reassortment events involving H7 in Africa (Figure 3). The H7 subtypes in the Americas group together as is also the case in Australia. The HPAIV conversions in Australia are part of a discrete monophyletic lineage that diverged from the Eurasian lineage (Figure 3). The first conversion event took place in Australia around 1976 and thereafter, all conversion form a single cluster with no introduction from other regions. Similarly, the H7 subtypes in Europe group together, and are only associated with conversion from LPAI to HPAI. The H7 HA was introduced into South Asia (Pakistan) around 1994, where it converted into a HP subtype.

## Discussion

The spatial and phylogenetic descriptive studies indicate that conversion and reassortment events, which are the two main evolutionary mechanisms by which a novel HPAI could emerge in the poultry systems, appear to show distinct geographical patterns.

The number of independent LPAI to HPAI conversion events is fairly low, even if some of these conversions lead to epidemics with very important consequences. The large majority of the detected conversions events took place in high-income countries, in poultry farms located within high poultry density areas. Out of the 41 conversion events documented, only 2 occurred in backyard rural flocks, and even those took place in regions of intensive poultry production (Italy 1997 and France 2015).

The surveillance and detection capacity can hardly be considered to be homogenous in space and time, and this may introduce a significant observational bias. So, a first hypothesis to explain the predominance of HPAI conversion in intensive poultry settings could be that this simply reflects the country’s surveillance capacity, but that other HPAI viruses could emerge in less intensive settings, and go extinct before being detected. Most HIC countries where HPAI conversions were described have regular and standardized surveillance and detection plans for sub-clinical LPAI and HPAI in poultry, and sometime in wild birds, too. In MICs, where poultry production systems are still transitioning toward more intensive production systems, only the large scale mortalities caused by HPAI in poultry would probably be noticed, often at times when the epidemic would have spread far and wide (e.g., Mexico, Pakistan, China). HPAI conversion in countries practicing vaccination as a means of control may be even more complicated to detect, as masking of clinical signs may confound the passive surveillance. The willingness to report an outbreak is also a factor that needs to be considered and commercial farms are more proactive when it comes to reporting (59), as compared to backyard farms. This willingness to report is more likely to be higher among farmers of HICs than in MICs too.

A second hypothesis is that HPAI conversions can only take place in intensive poultry rearing conditions with high contact rates, where certain mechanisms and conditions encountered in those systems aid conversions. Once a virus starts circulating in poultry, several variants are produced on account of antigenic drift and only the fittest variants persist. If host density and contact rates are low, the most virulent viruses may face a shortage of susceptible hosts and the chain of transmission may be broken. When the density and contact rates of immunologically naïve hosts increases, as it is encountered in a flock of intensively managed and unvaccinated broilers, for example (densities are typically >10 birds/m^2^ in a barn), the cost of gaining virulence decreases (the pathogen can kill its host quickly and still face a sufficiently large number of in-contact susceptible hosts to pursue its transmission in the population) and being highly pathogenic doesn’t limit transmission any longer (60, 61). Retrospective studies of LPAI and HPAI viruses from the same epidemic have shown that some of the key mutations that were present in the HPAI variants were also identified in the LPAI precursors, albeit at lower frequencies; and as the LP virus transmitted through the poultry population, it accumulated further mutations driven by a multitude of ecological drivers such as host, population and environmental changes, resulting in the conversion to a HPAI variant (62).

Another possible mechanism linking intensive poultry production systems to the evolution of virulence is the all-in/all-out practice, whereby entire cohorts of birds are managed simultaneously, and where the birds surviving an HPAI outbreak would be culled, which prevents the selection of natural resistance in the poultry host population. In contrast, in backyard poultry settings, birds that may have survived a local outbreak would possibly be used to restock with the possibility to select natural resistance genes, and mathematical models indicate that this may influence the evolution of virulence and host resistance (63).

The species composition also plays a role; there are differences in immune response to a LPAI and a HPAI infection within different species of poultry (64). In ducks, there is positive selection for genes that help to down regulate the immune response leading to tolerance for LPAI and HPAI infection. In chickens, immune response to LPAI is common (e.g., H9N2) but there is very little protective immune response to HPAI, and which may explain their increased susceptibility. Experimental studies in chicken show that the mutations like the C-terminal truncations at the non-structural 1 (NS1) protein occurring during the LP to HP conversion may help in increasing viral pathogenicity (65). Higher virulence can also be acquired with the insertion of polybasic amino acids in the HA cleavage site (HACS) motif (66). Another is the neuraminidase (NA) stalk region deletion that favour adaptation to terrestrial poultry (67), as well as increased respiratory shedding and virulence (68). However, the knowledge of selection pressures that lead to these mutations and subsequent switch can only be hypothesised and therefore requires further elucidation.

The role of vaccination in driving conversion also needs to be explored. Vaccination used for HPAI control following outbreaks in some of the countries may also favour the evolution of escape mutants that may convert into HP variants. Studies in Italy have shown the presence of certain mutations that were acquired after introduction of heterologous vaccination following the 1999 HPAI H7N1 epidemic (69) and the LPAI H7N3 outbreaks (70). Also in Mexico, antigenic drifts was observed in the HA gene of LP H5N2 AIV isolates over a period of time in vaccinated birds (11). In Egypt, a variant group of HPAI H5N1 viruses with increase in HA substitutions was isolated from vaccinated poultry following the implementation of vaccination in 2006 (71). These vaccination pressures may help in driving changes in the antigenicity that confer an evolutionary advantage and allowing re-infection of hosts, actually increasing respiratory shedding, onward transmission and a chance to change phenotype into HPAI.

In addition, the virus, host, and environment interactions driving conversions in Australia may be unique from other parts of the world. H7 viruses have not evolved antigenically over the last 30 years (72). Yet, there is remarkable diversity of the NA types (H7N2, H7N3, H7N4, H7N6, and H7N7) suggesting frequent reassortment with the wild bird system (73, 74). Australia and New Zealand lie at the southernmost edge of most of the migratory pathways with a climatic pattern quite different from that of the northern hemisphere with alternating wet and dry seasons that impacts the availability of food and breeding requirements for migrating Anseriformes (75), ducks, geese and swans, which represent one of the main reservoir of AI viruses. The Wallace Line, a well-known biogeography limit separating Australasia from Southeast Asia, may represent a barrier to the spread of AI into the Australian continent, as there is limited migration of Anseriformes birds across this line (76). Many species of shorebirds indeed do migrate across the Wallace Line to Australia after numerous stopovers in Asian countries with ongoing HPAI epizootics (77). However, the role of shorebirds in transmitting AI viruses to poultry over short and long distances is less well documented than from Anseriformes, and the LPAI viruses circulating in Australia may have limited genetic relatedness with those circulating in Asia.

The geographical distribution of HPAI reassortments is completely different, and points to the epidemiological conditions encountered in areas where HPAIV circulates endemically, the presence of a pre-existing HPAI being a necessary condition for the emergence of novel HPAI reassortants. The observational and surveillance intensity bias over space and time is likely to be much stronger for the dataset of HPAI reassortants than for the HPAI conversions. In a situation of HPAI endemicity where HPAI outbreaks are not systematically sampled and sequenced, active surveillance followed by sequencing is the main way of detecting a reassortant HPAI.

The epidemiological conditions promoting endemicity were documented in prior studies (78, 79). High duck density, with free-grazing in rice paddy fields are widespread in east Asia, and have been known to be associated with spread and persistence of HPAI (80). The production and marketing value chains associated with poultry production systems in middle income countries are characterized by many facilitators such as middlemen or aggregators who visit different farms, including some with low biosecurity, collect and redistribute multiple species of poultry and waterfowl, taking them to LBMs where there are ongoing possibilities of direct and indirect transmission (81). The LBMs of Asia have a high environmental load of AI viruses, low biosecurity and regulatory processes for minimizing contact points that foster virus survival and re-circulation and are key points of virus exchange and onward virus transmission (82).

Many countries in Asia and elsewhere have similar conditions, such as Bangladesh, Egypt, Indonesia, but yet had much fewer reassortants than China or Vietnam. In Bangladesh the detection rate of AIVs in the LBMs is quite high (24%) (83), the country has a sizeable population of ducks (though less intensively raised than China) and abundant waterfowl in the delta region between its two large rivers, which are also visited by migratory waterfowl from Europe and Central parts of Asia (84). Mixed rearing is common, with poultry and ducks raised together in a semi-scavenging system that allows contact with wild waterfowl (85). In Egypt, even though there has been ongoing circulation of HPAI H5N1 virus since 2006, the evolutionary trajectory has largely been of antigenic drift resulting in phenotypic variation, and few reassortants were reported. In Indonesia, apart from the reassortments that occurred in 2005–06, most of the evolution has been limited to point mutations (86) even though the country has numerous live-poultry markets with mixed species.

China and Vietnam implemented several national and regional level surveillance and epidemiological programs in the last decade, hence these were regions of increased sampling, and the higher frequency of reassortants found in these countries compared to other countries where similar risk factors prevail is likely influenced by this sampling bias.

However, this may not necessarily be the only explanation. We referenced the number of samples submitted to GenBank during each of the three time periods from the majority of countries reporting HPAI emergences (China, VietNam, Indonesia, Bangladesh, Egypt, and USA) (SI-Table S2), and found that the number of sequences submitted during the third time-period (2006–2015) is fairly homogenous relative to the chicken stock between all these countries (7) which suggests that the difference in reassortments cannot be fully explained by differences in sampling intensity.

Some epidemiological factors found in China, Hong Kong and Vietnam may differ from Bangladesh, Egypt and Indonesia. In China, Hong Kong and Vietnam, duck meat consumption is more popular, which translates into more intensive duck production systems and trade-related activities, which may also account for the diversity of subtypes of AIVs circulating within the LBMs. Particular subtypes/species combinations in the chicken/duck ecosystems are more prone to reassortment. A higher presence of particular AI subtypes in Anseriformes was described to be associated with higher reassortment rates as compared to Galliformes. Lu et al., (87) found that the H6 subtype that was more abundant in domestic ducks was associated with a higher rate of reassortment. They also found a positive correlation between subtypes with high reassortment rates and Anseriformes, i.e., waterfowl (87). In contrast, lower reassortment rates were observed in Galliformes.

China is also the world’s largest producer of swine and the presence of pigs at LBMs creates a higher risk of infection of LBM workers to poultry AIV and swine H1N1 viruses, additionally increasing the risk of emergence of novel HPAI (88). Bangladesh, Egypt and Indonesia are predominantly Muslim nations, and swine production is minimal. In China, other types of agricultural production, such as swine production and aquaculture have also been intensifying rapidly. The production practices unregulated with one industry’s waste being used as another industries input. For example, wastes from the swine and poultry industries are most often subjected to land disposal, which can lead to contamination of inland ground and surface water (89). Increasingly, poultry waste is also being used for land based aquaculture feeding in many countries (Little et al., 1996; Little and Edwards, 2003). Presence of remnant feed in poultry and pig wastes attracts waterfowl and wildbirds, which may also promote virus transmission if these waste disposal sites are located along migratory routes [such as in Guangdong, Southern China; (90)].

There is also an extensive wild bird interface in China that may explain the higher reassortment rates observed in east Asia, where large domestic ducks and geese populations are reared in mixed, free range settings that allows close contact of migratory and local waterfowls with ducks and geese, allowing for genetic exchange between viruses (91, 92). Several of the novel reassortants have had multiple gene segments from AI viruses of wild-bird origins (93).

Finally, in China, the rapid increase in HPAI reassortments and the increase in antigenic diversity also coincided with the time when mass-vaccination for HPAI control was implemented. Increase in antigenic drift and diversity promotes rapid antigenic evolution, which further complicates control by vaccination (94). It has not been conclusively proven that vaccination correlates with genetic reassortment, and even though vaccination protects poultry flocks from overt shedding of virus and clinical signs, subclinical infection and silent circulation in poultry does occur (95) and even leads to generation of reassortants as seen by the isolation of vaccine escape variants of HPAI H5N2 in China (96). There are also challenges associated with unregulated vaccine use in several countries that may lead to use of improperly inactivated or attenuated vaccines. In Taiwan, reassortant HPAI H5N2 viruses were isolated from outbreaks, which had HA and NA genes similar to the Mexican vaccine H5N2 strain, and other genes from enzootic AI H6N1 viruses (57).

The recent increase in the number of reassortments in east Asia, accompanied by rapid evolution and global spread of HPAI viruses deserves further investigations. In addition, the H5 HA that was almost exclusively associated with a monophyletic NA over the past decade had recently acquired the ability to combine with several NA subtypes (97) leading to generation of several H5Nx novel reassortants. The reason for this dramatic change in geographic and host range has been described in molecular terms (98). However, the underlying reasons or evolutionary pressures having favored these molecular changes are unknown. It has been hypothesized that recent changes in the spatial epidemiology of these reassortants HPAI H5Nx may have been associated with land use changes in northeastern China, having affected the spatial patterns of the wild – domestic interface (99).

Further studies on these aspects of differing duck densities and the associated rice-paddy-duck wild bird interface, leading to the risk of generation of genetic diversity will help to gain interesting insights into the generation of HPAI reassortants.

In conclusion, conversions and reassortments have distinct geographical and temporal patterns. The large majority of conversion events took place in intensively raised poultry conditions, but the precise role of this factor as compared to an observational bias would deserve a further quantitative investigation. The distribution of reassortments, that dominate in China and Vietnam, may be influenced by a surveillance and sequencing bias too, but one cannot exclude that other underlying causal factors favoring virus persistence, endemicity and co-circulation of several strains, in these transitioning poultry production systems may have been important too. There are still gaps in our knowledge of the drivers behind the differences in emergences of novel variants, either by conversion or reassortment. With the advent of deep sequencing methods, rapid progress is being made in the identification of molecular changes and mutations associated with evolution. Attempts should be made to monitor and link these changes to agro-ecological drivers of HPAI emergences, with an aim to adapt surveillance and control accordingly. Risk mitigation approaches to prevent conversions should consider the changing climate, land use, and poultry production scenarios, in order to adjust and target surveillance in changing wild-domestic bird interface for early detection of LP to HP conversion linked molecular signatures. Vaccination for prevention and control, should be accompanied with regular monitoring of post vaccinal sero-conversion, field virus surveillance and updating of vaccine strains. Most importantly, areas of intensifying poultry production need to be regulated to make sure that biosecurity and biosafety practices at all levels of the value chain reduce risk and are able to cope with the demands of intensification, and to avoid ongoing virus evolution.

## Author Contributions

MG, MSD and JA designed the study and reviewed methods, results and the manuscript. MSD, JA, SD, TVB, PL, collected data, analysed the data and wrote the manuscript. SM, DMC, SVD, GD, PL and MG interpreted the results, wrote and revised the manuscript.

## Conflict of Interest Statement

The authors declare that the submitted work was not carried out in the presence of any personal, professional or financial relationships that could potentially be construed as a conflict of interest.
