# Supplementary material for: Geographical and Historical Patterns in the Emergences of Novel Highly Pathogenic Avian Influenza (HPAI) H5 and H7 Viruses in Poultry
Source: Front Vet Sci. 2018 Jun 5;5:84. doi: 10.3389/fvets.2018.00084 (PMC5996087; doi:10.3389/fvets.2018.00084)
Supplement: Supplementary file 1 [file DataSheet1.DOCX]

***Data Sheet 1:*** **Details of Highly Pathogenic Avian Influenza subtype (HPAI) H5 and H7 conversions**

1959 -1995

Fifteen conversions from LPAI to HPAI were reported of which six occurred in Europe with four in UK and one each in Ireland and Germany (Figure-1 map, Table 1). Four conversion events occurred in Australia, and one each in the USA, Canada, South Africa, Mexico and Pakistan. These have been summarised in (Alexander and Brown, 2009). Notably, in the time line of events, in 1959, the first outbreak of HPAI H5N1 was reported from Scotland (Pereira et al., 1966) in the UK. In 1961, a first report of wild bird deaths caused by the HPAI H5 subtype (Becker, 1966) was reported where HPAI H5N3 caused large die-offs in common terns along the coast of South Africa. In Australia, from 1976 to 1994, four conversions of HPAI all caused by the H7 subtype were reported. In Germany, conversion of a LP H7N7 to a HP variant was reported from Leipzig, from a chicken farm. The unique aspect of this conversion event was that the HA of this virus isolate was only partially cleaved, (Röhm et al., 1996), in comparison to the other three isolates, which had a fully cleaved HA. All four virus isolates had a nearly identical HA gene sequence. Within Asia, only one HPAI conversion event was reported; an outbreak of H7N3 in northern Pakistan subsequent to acquisition of virulence following circulation as LP form in poultry (Naeem, 2009).

1996 - 2005

In this decade, eleven conversion events were documented. Two conversions from Italy and one from the Netherlands, were reported from Europe. In North America, one each from Canada and USA; one each from South Africa, Chile, and Australia and within Asia, one each from DPR Korea, Pakistan and China (Figure-1, Table 1). The earliest HPAI H5 conversion is believed to have occurred in Guangdong, China, in1996. The A/Goose/Guangdong/1/96 (H5N1) virus (hereafter called Gs/Gd virus) apparently co-circulated in Southern China within domestic ducks, acquiring the ability to transmit from the avian aquatic reservoirs to terrestrial poultry (Guan et al., 2002a), causing high mortality in domestic geese farms (Xu et al., 1999). In 1997, HPAI H7N4 was also reported from New South Wales in Australia (Selleck et al., 2003). In Italy, two conversions of HPAI were reported in 1997. The first involved H5N2 in 1997 in north-eastern Italy (Capua et al., 1999) within an area of migratory flyways and the second caused by a H7N1 in 1999, following a prolonged and widespread LPAI circulation in the northern part of Italy (Capua et al., 2000). In 2002, HPAI H7N3 was reported in Chile, believed to be the first isolation of HPAI in South America. This HP H7N3 virus was believed to have converted from a LP variant as a likely result of recombination between HA and NP gene of the virus (Suarez et al., 2004). In 2003, in Netherlands, H7N7 emerged in a dense poultry rearing area, originating from an LPAI outbreak in a nearby poultry shed (Stegeman et al., 2004). In Canada, HPAI H7N3 was reported in Fraser Valley, caused by mutation from a LP to HP variant, associated with insertion of seven amino acids in the HA gene conferring high pathogenicity (Hirst et al., 2004). In 2003, a LPAI H7N3 subtype was reported from southern Pakistan, where it circulated undetected in commercial poultry for a few months before mutating into a HP variant (Abbas et al., 2010).

In USA, HPAI H5N2 occurred in Texas in 2004 and was the first highly pathogenic strain reported after 20 years. This H5N2 was a highly unusual as it met the molecular criteria of OIE for classification as HP, but caused only a low pathogenic clinical disease upon experimental inoculation in chickens (Lee et al., 2005b). Similarly, a HP H5N2 was isolated for the first time from farmed ostriches in forty-three years, in South Africa in 2004. This virus also had the molecular characteristics of a HP virus, but exhibited low pathogenicity in the ostriches (Abolnik et al., 2009).

2006 - 2015

Thirteen conversion events were reported. In Europe, Germany and UK reported two events of conversion each, and Spain, Italy and France, one each. Australia had two conversion events. In North America, Canada, and the USA reported one each (Figure-1, Table 1) and South Africa reported one conversion event. Mexico also reported a conversion event. In 2007, in Canada, in Saskatchewan, a low pathogenic precursor H7N3 virus evolved into a HP variant following spill over from wild birds and subsequent undetected circulation in (broiler breeder) poultry (Berhane et al., 2009). In 2008, HPAI H7N7, was reported from Oxfordshire, UK (Defra, 2008) and was the first report of a HPAI H7 conversion. In 2009, HPAI H7N7 emerged in Spain for the first time in a farm located close to a reservoir wetland (Iglesias et al., 2010). In 2011, HPAI H5N2 emerged in ostriches in South Africa with lack of genotypic relatedness to the earlier 2004 and 2006 outbreaks, and a wild duck LP progenitor virus was established (Abolnik et al., 2012). In 2012, HPAI emerged in Australia and Mexico. In Australia, H7N7 HPAI was reported from Maitland in New South Wales in a free-range layer chicken flock located close to a dam frequented by wild birds (FAO, Empres-i). In Mexico, a low pathogenic H7N3 virus acquired an extended cleavage motif by recombination with host 28S rRNA and not from the viral genome. This was a novel instance where such a cleavage motif had been acquired by such means naturally, which had only been observed in laboratory strains earlier (Maurer-Stroh et al., 2013)

In 2013, two conversions of HPAI were reported. Italy reported an outbreak of H7N7 from the Po river delta area, where contact of free-range hens with wild waterfowl led to the conversion of LP into a HP strain. The virus was found to be genetically related to the central European and Italian LP H7N7 viruses (Bonfanti et al., 2014). Australia reported an outbreak of H7N2 caused by the transmission of a low pathogenic virus from a wild birds to poultry which then turned highly pathogenic as it spread among the farmed chickens (FAO, Empres-i). In 2014, HPAI H5N8 belonging to H5 clade 2.3.4.4 emerged in Germany in a turkey flock, believed to have been carried to Germany via subclinically infected migratory birds (Harder et al., 2015). In 2015, H7N7 HPAI emerged in UK following mutation from a LP form to a HP variant in a laying egg farm. The likely source of the virus was believed to be wild birds visiting waterbodies on farm premises (Defra, 2015). Germany reported the emergence of HPAI H7N7 shortly afterwards. The layer farm was located very near another farm that had reported LPAI H7N7 a month ago (OIE, 2015). In end-2015, France reported three new strains of HPAI in quick succession; H5N1, H5N2, and H5N9 that belonged to the Eurasian lineage, and not to the Gs/Gd lineage. The conversion of LP to HPAI H5N1 was reported from a backyard chicken farm and the other two viruses were reported from duck fattening farms. The HA genes of these three viruses were very similar (Briand et al., 2017), and the hypothesis is that there was a passage from a low pathogenic HA cleavage site into a HP one (H5N1), based on at least five nucleotide substitutions in the HA cleavage site, and not insertions as is the usual case. In Dec 2015, a first instance of a LPAI H7N8 converting into a HPAI variant following undetected circulation in poultry, leading to an outbreak in a turkey flock in Indiana, USA (Killian et al., 2016).

An observation of the host species and production, systems involved shows that on a species level, the LP to HP conversions have been most associated with chicken production (27 times), followed by turkey rearing (8), geese and ostrich (2) and once observed in ducks and wild terns. Conversions have been largely dominant in commercial poultry production systems. Commercial chicken production systems have been affected twenty-five times, followed by commercial turkey farms that have been affected eight times. Conversion in commercial ostrich farms has been reported from South Africa twice. Commercial geese farms have also been affected twice. There has been only a single report of commercial duck farm being associated with LP to HP conversion, in Europe. Backyard farms have reported in only two instances of LP to HP conversion, both in Europe. In 1997, in Italy, a HPAI H5N2 was reported in backyard chickens in an area in the northeast of Italy. In 2015, HPAI influenza A (H5N1) was detected in birds at a backyard broiler farm in France. The only instance of a conversion being documented in wild birds was the large die-off of wild terns caused by HPAI H5N3 in 1961, off the coast of South Africa (Becker, 1966)

There is evidence of a direct interface with wild birds in nineteen of total conversion events. This includes proximity to areas inhabited by wild birds, areas of overlap with migratory bird flyways and direct links established with wild bird sequences through phylogenetic analyses. In the remaining conversion events, mutation into increased pathogenicity has occurred as a result of undetected prolonged circulation of a LP variant in poultry even though the initial spill over from wild migratory or resident birds may have occurred at some point.

**References:**

Abbas, M.A., Spackman, E., Swayne, D.E., Ahmed, Z., Sarmento, L., Siddique, N., Naeem, K., Hameed, A., Rehmani, S., 2010. Sequence and phylogenetic analysis of H7N3 avian influenza viruses isolated from poultry in Pakistan 1995-2004. Virol J 7, 1–10. doi:10.1186/1743-422X-7-137

Abolnik, C., Londt, B.Z., Manvell, R.J., Shell, W., Banks, J., Gerdes, G.H., Akol, G., Brown, I.H., 2009. Characterisation of a highly pathogenic influenza A virus of subtype H5N2 isolated from ostriches in South Africa in 2004. Influenza Other Respir. Viruses 3, 63–68. doi:10.1111/j.1750-2659.2009.00074.x

Abolnik, C., Olivier, A.J., Grewar, J., Gers, S., Romito, M., 2012. Molecular analysis of the 2011 HPAI H5N2 outbreak in ostriches, South Africa. Avian Dis. 56, 865–879. doi:10.1637/10171-041012-Reg.1

Alexander, D.J., Brown, I.H., 2009. History of highly pathogenic avian influenza. Rev. Sci. Tech. Int. Off. Epizoot. 28.

Becker, W.B., 1966. The isolation and classification of Tern virus: influenza A-Tern South Africa--1961. J. Hyg. (Lond.) 64, 309–320.

Berhane, Y., Hisanaga, T., Kehler, H., Neufeld, J., Manning, L., Argue, C., Handel, K., Hooper-McGrevy, K., Jonas, M., Robinson, J., Webster, R.G., Pasick, J., 2009. Highly pathogenic avian influenza virus A (H7N3) in domestic poultry, Saskatchewan, Canada, 2007. Emerg. Infect. Dis. 15, 1492–1495. doi:10.3201/eid1509.080231

Bonfanti, L., Monne, I., Tamba, M., Santucci, U., Massi, P., Patregnani, T., Piccolomini, L.L., Natalini, S., Ferri, G., Cattoli, G., Marangon, S., 2014. Highly pathogenic H7N7 avian influenza in Italy. Vet. Rec. vetrec–2013–102202. doi:10.1136/vr.102202

Briand, F.-X., Schmitz, A., Ogor, K., Le Prioux, A., Guillou-Cloarec, C., Guillemoto, C., Allée, C., Le Bras, M.-O., Hirchaud, E., Quenault, H., Touzain, F., Cherbonnel-Pansart, M., Lemaitre, E., Courtillon, C., Gares, H., Daniel, P., Fediaevsky, A., Massin, P., Blanchard, Y., Eterradossi, N., van der Werf, S., Jestin, V., Niqueux, E., 2017. Emerging highly pathogenic H5 avian influenza viruses in France during winter 2015/16: phylogenetic analyses and markers for zoonotic potential. Euro Surveill 22. https://doi.org/10.2807/1560-7917.ES.2017.22.9.30473

Capua, I., Marangon, S., Selli, L., Alexander, D.J., Swayne, D.E., Pozza, M.D., Parenti, E., Cancellotti, F.M., 1999. Outbreaks of highly pathogenic avian influenza (H5N2) in Italy during October 1997 to January 1998. Avian Pathol. 28, 455–460. doi:10.1080/03079459994470

Capua, I., Mutinelli, F., Marangon, S., Alexander, D.J., 2000. H7N1 avian influenza in Italy (1999 to 2000) in intensively reared chickens and turkeys. Avian Pathol. 29, 537–543. doi:10.1080/03079450020016779

DEFRA, 2015. H7N7 HPAI in Germany.pdf [WWW Document]. URL https://www.gov.uk/government/uploads/system/uploads/attachment_data/file/450042/poa-hpai-germany-h7n7-072015.pdf (accessed 3.1.16).

Forsyth, W., Grix, D., Gibson, C., 1993. Diagnosis of highly pathogenic avian influenza in chickens: Bendigo 1992. Aust. Vet. J. 70, 118–119. doi:10.1111/j.1751-0813.1993.tb03294.x

Guan, Y., Peiris, J.S.M., Lipatov, A.S., Ellis, T.M., Dyrting, K.C., Krauss, S., Zhang, L.J., Webster, R.G., Shortridge, K.F., 2002. Emergence of multiple genotypes of H5N1 avian influenza viruses in Hong Kong SAR. Proc. Natl. Acad. Sci. U. S. A. 99, 8950–8955. doi:10.1073/pnas.132268999

Harder, T., Maurer-Stroh, S., Pohlmann, A., Starick, E., Höreth-Böntgen, D., Albrecht, K., Pannwitz, G., Teifke, J., Gunalan, V., Lee, R.T.C., Sauter-Louis, C., Homeier, T., Staubach, C., Wolf, C., Strebelow, G., Höper, D., Grund, C., Conraths, F.J., Mettenleiter, T.C., Beer, M., 2015. Influenza A(H5N8) Virus Similar to Strain in Korea Causing Highly Pathogenic Avian Influenza in Germany. Emerg. Infect. Dis. 21, 860–863. doi:10.3201/eid2105.141897

Hirst, M., Astell, C.R., Griffith, M., Coughlin, S.M., Moksa, M., Zeng, T., Smailus, D.E., Holt, R.A., Jones, S., Marra, M.A., Petric, M., Krajden, M., Lawrence, D., Mak, A., Chow, R., Skowronski, D.M., Tweed, S.A., Goh, S., Brunham, R.C., Robinson, J., Bowes, V., Sojonky, K., Byrne, S.K., Li, Y., Kobasa, D., Booth, T., Paetzel, M., 2004. Novel Avian Influenza H7N3 Strain Outbreak, British Columbia. Emerg. Infect. Dis. 10, 2192–2195. doi:10.3201/eid1012.040743

Horimoto, T., Rivera, E., Pearson, J., Senne, D., Krauss, S., Kawaoka, Y., Webster, R.G., 1995. Origin and Molecular Changes Associated with Emergence of a Highly Pathogenic H5N2 Influenza Virus in Mexico. Virology 213, 223–230. doi:10.1006/viro.1995.1562

Iglesias, I., Martínez, M., Muñoz, M.J., De La Torre, A., Sánchez-Vizcaíno, J.M., 2010. First Case of Highly Pathogenic Avian Influenza in Poultry in Spain. Transbound. Emerg. Dis. 57, 282–285. doi:10.1111/j.1865-1682.2010.01145.x

Kawaoka, Y., Naeve, C.W., Webster, R.G., 1984. Is virulence of H5N2 influenza viruses in chickens associated with loss of carbohydrate from the hemagglutinin? Virology 139, 303–316.

Killian, M.L., Kim-Torchetti, M., Hines, N., Yingst, S., DeLiberto, T., Lee, D.-H., 2016. Outbreak of H7N8 Low Pathogenic Avian Influenza in Commercial Turkeys with Spontaneous Mutation to Highly Pathogenic Avian Influenza. Genome Announc. 4, e00457–16. doi:10.1128/genomeA.00457-16

Lee, C.-W., Swayne, D.E., Linares, J.A., Senne, D.A., Suarez, D.L., 2005. H5N2 Avian Influenza Outbreak in Texas in 2004: the First Highly Pathogenic Strain in the United States in 20 Years? J. Virol. 79, 11412–11421. doi:10.1128/JVI.79.17.11412-11421.2005

Maurer-Stroh, S., Lee, R.T., Gunalan, V., Eisenhaber, F., 2013. The highly pathogenic H7N3 avian influenza strain from July 2012 in Mexico acquired an extended cleavage site through recombination with host 28S rRNA. Virol J 10, 139. doi:10.1186/1743-422X-10-139

McNulty, M.S., Allan, G.M., McCracken, R.M., McParland, P.J., 1985. Isolation of a highly pathogenic influenza virus from Turkeys. Avian Pathol. 14, 173–176. doi:10.1080/03079458508436216

OIE, 2015. The World Animal Health Information System: OIE - World Organisation for Animal Health [WWW Document]. URL http://www.oie.int/animal-health-in-the-world/the-world-animal-health-information-system/the-oie-data-system/ (accessed 2.1.17).

Pereira, H.G., Lang, G., Olesiuk, O.M., Snoeyenbos, G.H., Roberts, D.H., Easterday, B.C., 1966. New antigenic variants of avian influenza A viruses. Bull. World Health Organ. 35, 799–802.

Röhm, C., Süss, J., Pohle, V., Webster, R.G., 1996. Different Hemagglutinin Cleavage Site Variants of H7N7 in an Influenza Outbreak in Chickens in Leipzig, Germany. Virology 218, 253–257. doi:10.1006/viro.1996.0187

Selleck, P.W., Arzey, G., Kirkland, P.D., Reece, R.L., Gould, A.R., Daniels, P.W., Westbury, H.A., 2003. An Outbreak of Highly Pathogenic Avian Influenza in Australia in 1997 Caused by an H7N4 Virus. Avian Dis. 47, 806–811.

Stegeman, A., Bouma, A., Elbers, A.R.W., Jong, M.C.M. de, Nodelijk, G., Klerk, F. de, Koch, G., Boven, M. van, 2004. Avian Influenza A Virus (H7N7) Epidemic in The Netherlands in 2003: Course of the Epidemic and Effectiveness of Control Measures. J. Infect. Dis. 190, 2088–2095. doi:10.1086/425583

Suarez, D.L., Senne, D.A., Banks, J., Brown, I.H., Essen, S.C., Lee, C.-W., Manvell, R.J., Mathieu-Benson, C., Moreno, V., Pedersen, J.C., Panigrahy, B., Rojas, H., Spackman, E., Alexander, D.J., 2004. Recombination Resulting in Virulence Shift in Avian Influenza Outbreak, Chile. Emerg. Infect. Dis. 10, 693–699. doi:10.3201/eid1004.030396

Xu, X., Subbarao, K., Cox, N.J., Guo, Y., 1999. Genetic Characterization of the Pathogenic Influenza A/Goose/Guangdong/1/96 (H5N1) Virus: Similarity of Its Hemagglutinin Gene to Those of H5N1 Viruses from the 1997 Outbreaks in Hong Kong. Virology 261, 15–19. doi:10.1006/viro.1999.9820

Wells R.J.H. (1963). An outbreak of fowl plague in turkeys. Veterinary Record, 75,.783-786. 39
