# Supplementary material for: Geographical and Historical Patterns in the Emergences of Novel Highly Pathogenic Avian Influenza (HPAI) H5 and H7 Viruses in Poultry
Source: Front Vet Sci. 2018 Jun 5;5:84. doi: 10.3389/fvets.2018.00084 (PMC5996087; doi:10.3389/fvets.2018.00084)
Supplement: Supplementary file 2 [file DataSheet2.DOCX]

**Data Sheet 2: Details of Highly Pathogenic Avian Influenza subtype (HPAI) H5 and H7 reassortments**

The details of all the HPAI reassortments events are provided in Table 1 SI. Temporally as done earlier, we studied the reassortments in three time periods. Up to the year 1995, no HPAI reassortments were reported in literature.

1996-2005: This decade of HPAI was the period in which the H5N1 subtype spread, causing devastating epidemics in poultry across Asia. Until this period, the emergence of HPAI in earlier outbreaks was primarily a case of a LP virus acquiring mutations and converting to a highly pathogenic phenotype for chickens (Kawaoka et al., 1984) (García et al., 1996) (Perdue, 1997). However, in this decade, several reassortment events occurred leading to generation of multiple genotypes of H5N1, and rapid evolution of the virus characterised by changes in pathogenicity and host range.

A total of forty-five reassortments were documented; all HPAI reassortments during this period occurred in the H5N1 subtype. Southern China was the centre of the reassortment events up to 2003 (east Asia as seen in Figure-2). In March 1997, an outbreak of HPAI H5N1 was reported from poultry farms in north western Hong Kong. Subsequently, the pandemic nature of the virus was unveiled with the deaths of six humans caused by a HPAI H5N1 virus (Claas et al., 1998) ; (Subbarao et al., 1998). The evolution of this pandemic virus was traced back to the progenitor virus A/Goose/Guangdong/1/96 (H5N1)(GSGD/1/96) isolated from the outbreak in in geese in Guangdong, China.

Retrospective phylogenetic studies conducted after the Hong Kong pandemic showed that the (Gs/Gd/1/96) likely reassorted with other avian viruses yielding a triple reassortant, with the HA gene being highly homologous with the A/goose/Guangdong/1/96 (H5N1) and the other internal gene segments acquired from the H9N2 and H6N1 circulating in quails in the live bird poultry markets of Hong Kong (Xu et al., 1999)(Guan et al., 1999).

In 2000-01, reassortment was detected in geese and ducks imported from mainland southern China to Hong Kong during the surveillance of live-bird markets (LBMs) in Hong Kong. Studies revealed that even though the HA gene belonged to the prescursor Gs/Gd/96-like virus, their internal protein genes had been acquired through genetic reassortment with other waterfowl avian influenza viruses (Guan et al., 2002b). Five different (A to E) genotypes were generated by such reassortments (Guan et al., 2002a). Meanwhile in China, these novel reassortants were detected in five provinces; Fujian, Guangdong, Guangxi, Jiangsu and Zhejiang, with the earliest reassortant detected in Hubei in 1997. Two major multiple reassortment events occurred during 2001-02, leading to the generation of several different genotypes (Duan et al., 2008). The first wave of reassortments led to the generation of genotype B (B, B1 and B2) followed by generation of Genotype W. The second multiple reassortment event took place in Hong Kong between 2001-02, where genotype E reassorted with other aquatic AI viruses to yield genotypes X0-X3.

In 2002, genotype B reassorted to yield Genotype Z, in Guangxi, which further reassorted with other aquatic viruses to yield genotypes G and V (Duan et al., 2008) . Genotype A, C, D, and E and their progenitor, the *Gs/Gd* virus were no longer detected. By 2001, the reassortants were discovered from eight provinces in China, with the addition of Shanghai, Hebei, and Henan. By 2003, genotype X had disappeared, and by 2005, another major reassortment event led to the appearance of the new genotype V from genotype Z.

In late 2002, an outbreak of HPAI H5N1 was reported from wild migratory birds and local waterfowl in two parks of Hong Kong. The virus isolated was a reassortant virus that had acquired the ability to cause severe systemic infection in ducks (Sturm-Ramirez et al., 2004). This was the first lethal outbreak of H5N1 reported from wild birds since 1961. This virus was similar to genotype Z, except that it had a 20 amino acid deletion in the NA, and was called as genotype Z+ (Guan et al., 2004). In 2003, another human death due to HPAI H5N1 was reported from Hong Kong after the man travelled to Fujian in China. The virus was identical to genotype Z+ that had caused the wild bird outbreaks(Guan et al., 2004). Another reassortant, genotype Y was detected from outbreaks in chicken farms during this period.

In 2003, a novel HPAI H5N1 multiple reassortant was isolated from swine in Shandong province in China (Shi et al., 2008) that had H9N2-like M and NS genes,as well as unknown NP, PA and PB1 genes. In 2004, a new reassortant genotype was detected in tree sparrows in Pingyu County, Henan, China (Kou et al., 2005). In 2005, a massive outbreak caused by HPAI H5N1 was reported from wild birds at Qinghai Lake in Western China. Four different genotypes (A to D) were involved, but one unique reassortant isolate of genotype B had four genes from wild bird viruses along with a PB2 gene from genotype A viruses (Chen et al., 2006; )(Li et al., 2010).

By 2005, genotype Z was geographically becoming dominant towards southeast Asia and the HA gene was further evolving into generating first and second order clades 1 and 2.1, whereas genotype Z was being replaced in southern China and neighboring countries by a genotype V sub lineage, clade 2.3.4 [previously called Fujian like, (Smith et al., 2006)]. In all, from China and Hong Kong, during 1996-2005, a total of forty-two reassortant H5N1 viruses were generated with a dramatic increase in genotypic diversity. From 2003 onwards, these viruses spread geographically, simultaneously accumulating antigenic drift in the HA gene, leading to the generation of various clades. Spread to Korea, Vietnam, Japan, Thailand, China, Cambodia, Lao PDR, Indonesia, Hong Kong and Malaysia was reported from outbreaks in poultry during 2003-04. A reassortant HP H5N1 genotype was generated in Vietnam in 2005, that was likely generated as a result of reassortment of the circulating genotype with the newly introduced clade 2.3.2 viruses in northern Vietnam (Wan et al., 2008). Interlineage reassortment was also reported from Indonesia, with reassortant viruses isolated from human outbreaks (Lam et al., 2008).

HPAI H5N1 reassortment was reported in 2003, from an outbreak in DPR Korea. The Korean isolate had the HA and NA from the Gs/Gd lineage, but had a unique PA gene (from a duck H9N1 virus), indicating reassortment (Lee et al., 2005a).

2006-2015: This period was characterized by not only an increase in virus evolution, but also in the geographical niche of the virus. The maximum number of reassortments occurred in Asia (73) indicating the intensity of virus evolution in this region. Five reassortments were reported from Africa and three from North America. In Asia, Vietnam had the maximum number (35) of reassortments followed by China (25), Taiwan and Bangladesh and Korea (3), and Lao PDR, Indonesia and Kazakhastan with one each. In North America, one reassortment event was reported from USA, Canada, and Mexico each. Africa had a total of five reassortments, with four of them from Nigeria and the remaining one from South Africa. No reassortments were reported from Europe and Australia.

During 2005, HPAI caused by H5N1, started spreading westwards with outbreaks being reported from poultry in Russia, Kazakhstan and wild birds in Mongolia. Spread to several countries within Asia, eastern Europe and northern Africa was reported and by mid 2006, the Asian HPAI H5N1 had spread to 31 countries (OIE, 2015). In 2006, in a wild bird outbreak in Qinghai Lake caused by HPAI H5N1, a reassortant isolated from bar headed goose. The European-Middle Eastern-African (EMA) clade evolved from the H5N1 viruses of Qinghai and further evolved into 3 clades (EMA1-3) (Salzberg et al., 2007).

In 2006, within the EMA lineage circulating in the European and African countries, reassortment was reported in Nigeria in 2006, where a 4:4 reassortant was isolated (Salzberg et al., 2007). Other than these reassortments, HPAI H5N2 reassortment was reported from Western Cape, South Africa, from an outbreak in ostriches. This virus had internal genes completely different from the earlier H5N2 indicating reassortment in other aquatic hosts (Abolnik, 2007). In March 2006, deaths of mute swans were reported near Kazakhstan, from which a H5N1 virus was isolated that was not related to the earlier clade 2.2 Qinghai genotype. This H5N1 virus was a reassortant virus likely to have been derived from the circulating H5N1 viruses in Japan and Russian far east that was brought into Kazakhstan by the Black Sea migratory routes (Tabynov et al., 2014).

Major evolutionary events were reported from Asia during this period. During 2006, in China, the genotype V evolved into genotype V2 by incorporating novel PB1 and PB2 genes through reassortment. This genotype V2 later went on to become established in Vietnam and Thailand. Vietnam was the centre of a majority of reassortment events during 2007-2010 where several novel genotypes (34) were identified some of which persisted for long periods, whereas others were transient. Genotype VN6, 7, and 9 were isolated from North Viet Nam whereas genotype VN8 was isolated from ducks in Southern Viet Nam (Wan et al., 2008). In 2007, these were the genotypes VN 29-32, VN38 and VN41; in 2008, genotypes VN19-22, VN34, and VN42-44; in 2009- VN17-18, VN27, VN33, and VN 39; and in 2010- VN14, VN25-26, and VN35-37 (Nguyen et al., 2012). Reassortant virus from cats in Indonesia was also isolated, belonging to a sub-lineage of genotype Z (Lam et al., 2008)

Apart from Vietnam, reassortment in HPAI H5N1 was also reported from Nigeria in 2007, where a 2:6 EMA1/EMA2 reassortant was identified (Monne et al., 2008). Additional reassortants were identified in Nigeria that were ressortants derived from the earlier reassorted sub-lineages indicating developing endemicity in the region (Owoade et al., 2008).

From 2008-2010, apart from Vietnam, all other ressortments were reported from China. Until this time, most of the reassortments were being documented with the H5N1 HPAI viruses with the NA gene being derived from the Gs/Gd like viruses. The period after 2009 saw a dramatic increase in genetic diversity of H5 viruses with several novel reassortants bearing the backbone of Gs/Gd-like H5, but having the NA of other subtypes. In 2008, HPAI H5N5 viruses were isolated from ducks in LBM in southern China, which had NA from other origins. These were multiple reassortants among the H5N1, H6N5, and other H5N1, H5N2, and H6N2 subtypes. The M gene was derived from human H5N1 strain (Liu et al., 2013) indicating reassortment between human and aquatic waterfowl viruses. Additionally, novel H5N5 viruses were recovered from apparently healthy mallard ducks during LBM surveillance having HA gene belonging to clade 2.3.4 (Gu et al., 2011) with the NA gene most likely derived from H6N5 viruses.

In 2009, novel H5N5 and H5N8 viruses were isolated and from quail, geese, and ducks during surveillance in LBMs in eastern China. These viruses were reassortants of H5N1 with the PB1, PB2, and NA genes derived from unknown viruses (Zhao et al., 2013). In 2010-11, four novel reassortant HPAI H5N2 viruses were isolated from poultry vaccinated with a bivalent (H5N1 and H9N2) influenza vaccine during LBM surveillance in eastern China. Prior to this, in Asia, H5N2 had always been isolated in a LP form. Two of these viruses belonged to clade 2.3.4, and two belonged to clade 7, with the chicken isolate having internal genes from H9N2 viruses (Zhao et al., 2012). The clade 7 reassortant from duck, interestingly was low pathogenic in chickens and ducks, even though it had a HA cleavage signature motif of a highly pathogenic variant (Zhao et al., 2012)

In 2012, further reassortments in HPAI H5N1 viruses were reported from Vietnam. Genotype VN46 and VN47 were generated by reassortment between clade 2.3.2.1-A and 2.3.2.1-B viruses. Genotype VN48 was derived from reassortment between the HA of clade 2.3.2.1-C virus, which had the NA and other internal genes from 2.3.2.1-A and 2.3.2.1-B viruses (Creanga et al., 2013). Taiwan also reported widespread outbreaks from HPAI H5N2 in 2012, wherein the H5N2 virus was related to a Mexican vaccine strain virus, suggesting incomplete attenuation of vaccine strain. Thereafter, this virus had possibly undergone multiple reassortments with the enzootic human H6N1 viruses (C.-C. D. Lee et al., 2014).

Reassortment was reported for the first time in Bangladesh in 2012, even though it has one of the highest numbers of outbreaks of HPAI in poultry. A novel H5N1/H9N2 7:1 reassortant having PB1 gene from co-circulating H9N2 virus was identified (Monne et al., 2013). In Mexico, a HPAI H7N3 of wild bird origin, unrelated to the earlier outbreaks in North America was the reported from widespread poultry outbreaks in Jalisco, 2012. The virus was a complicated reassortant with five genes from wild birds migrating along the central North American flyway and the others from wildbirds along the western North American flyway (Lu et al., 2014a).

In 2013, for the first time, reassortment was detected in viruses isolated from human cases in Vietnam. Until this time, all human cases had been caused by clade 1 H5N1 viruses having a similar genetic makeup since 2003. The 2013 reassortant genotype VN49 had a clade 1.1.2 HA and NA, but internal genes derived from clade 2.3.2.1a viruses (Thor et al., 2015). Also in eastern China, in 2013, reassortants belonging to subtype H5N8 were isolated from domestic ducks, that had genes from H11N9 and H4N2 viruses (Wu et al., 2014). Additionally, a reassortant H5N2 was isolated from a chicken in Jiangsu, which had the backbone of the earlier reassorted H5N2 (Zhao et al, 2012), and had acquired the matrix protein gene from a H5N1 clade 7.2 virus (Mi et al., 2013).

From 2014 onwards, outbreaks caused by novel reassortants were reported from diverse geographic areas. A majority of these were inter subtype reassortants, with novel HP H5Nx constellations. These included H5N6, H5N8, H5N3, H5N2, and H5N9 in addition to reassortant H5N1. All the reassortants belonged to clade 2.3.4 and the extent of genomic divergence was such that these had to be classified into fourth order clade of 2.3.4.4. (<http://www.who.int/influenza/gisrs_laboratory/h5_nomenclature_clade2344/en/>).

In 2014, a novel reassortant HPAI H5N6 was reported from Lao PDR, that had genes from H5 clade 2.3.2.1b, clade 2.3.4.4, and influenza A H6N6 viruses (Wong et al., 2015). Novel HPAI H5N8 emerged in South Korea causing mortalities in a breeder duck farm located near the Donglim reservoir where multiple mortalities in Baikal teals had been reported. The viruses isolated belonged to the novel H5 clade 2.3.4.4. One of the viruses was a reassortant of H5N8 virus isolated earlier from Jiangsu, and a H11N9 virus that donated the PB2 and NS genes. The other two reassortants were H5N8 having PB1, PA, M and NS gene homologous to H5N2 viruses from eastern China (Lee et al., 2014). A second reassortant H5N8 was isolated from surveillance of waterfowls in Pungse River in South Korea. The H5N8 virus was a reassortant with PB2, HA and NP genes from a H5N1-like virus, NA from H5N8, and the remaining gene segments from a H4N2 virus (Ku et al., 2014).

In China, novel reassortant clade 2.3.4 H5N1 was reported from an outbreak in chicken egg farms in Yunnan province. The HA of the virus was similar to the H5N6 isolate from Lao PDR, and the NA was related to the Vietnamese H5N1 isolates. The remaining internal genes were similar to H5N2 from Jiangxi (Hu et al., 2015). Thereafter, a never before reported H5N3 was isolated from a live bird market in Changsha, Hunan, China (EMPRES-i) belonging to H5 clade 2.3.4.4.

After November 2014, widespread geographic spread of these novel reassortants began to other parts of Asia, Europe and North America. The novel H5N8 clade 2.3.4.4 viruses caused the first outbreak by a Eurasian lineage HPAI virus in Canada, in November 2014. The causative virus was reassortant HPAI H5N2 that possessed gene segments derived from the Eurasian HPAI H5N8 viruses and indigenous North American waterfowl viruses (Pasick et al., 2015). The Eurasian H5N8 viruses were also isolated from wild waterfowl in British Columbia, Canada, and from dead falcons in Washington, USA, indicating that wild birds were the likely source of the virus into North America, where it reassorted with the indigenous lineages. In the USA, in addition to the Eurasian H5N8, a reassortant H5N1 was also isolated from a green winged teal in 2015. This reassortant virus had 4 genes from the Eurasian H5N8 and the other four from the low pathogenic north American viruses (Torchetti et al., 2015). Novel reassortments were also reported from France from duck fattening farms. The novel HPAI H5N2 was reported from a commercial duck fattening farm, whereas the H5N9 was reported from a small (500 birds) duck fattening farm. These viruses had very similar HA gene and cleavage site, but the internal genes of these viruses were quite different. It is likely that the HPAI H5N2 and the H5N9 viruses arose from different reassortment events (Briand et al., 2017)

The novel H5N8 in 2015, spread into Taiwan, where it reassorted with other AI viruses to yield novel reassortant viruses of H5N8, H5N2, and H5N3 subtypes causing the loss of 3.2 million birds within six months (Huang et al., 2016).

In China, in 2015, a novel H5N9 virus that was a reassortant of H5N1, H7N9 and H9N2 subtypes, was isolated from LBMs in Hangzhou, China. The HA gene was derived from clade 2.3.2.1 Vietnamese viruses, whereas the NA was derived from the human infecting LP H7N9 viruses from China (Yu et al., 2015). In Nigeria, high mortality in backyard poultry was reported from Lagos State in January 2015. The H5N1 virus was a intersubtype reassortant belonging to HA clade 2.3.2.1c and was similar to the viruses from China and Vietnam, and also to the H5N1 virus that had caused human infection in a Canadian resident who had traveled to China. The PB2 gene segment belonged to the Asian H9N2(Monne et al., 2015). Additionally, a clade 2.3.2.1c reassortant HPAI H5N1 from a migratory bird die-off in Sanmenxia reservoir having a H9N2 derived PB2 gene was isolated from whooper swans (Bi et al., 2015b).

Reassortments in the HPAI H5 viruses also increased the likely pandemic virus pool. Earlier human infections had been reported with only H5N1 and H5N2 subtypes. In Feb 2014, a first case of human infection caused by a reassortant HPAI H5N6 virus was isolated from Changsha, China. The virus was a triple reassortant with a H5N1 HA, a H10N6 NA and six internal genes from a duck H5N2 virus isolated in Jiangxi (Zhang et al., 2016) . Thereafter, another reassortant HPAI H5N6 was isolated from a human patient in Sichuan province. Phylogenetic analysis showed a causative triple reassortant AI virus having HA from clade 2.3.4.4 H5 viruses, a H6N6 virus derived NA, and six internal genes belonging to clade 2.3.2.1 H5 viruses (Pan et al., 2016). A HPAI H5N6 was also isolated chickens during LBM surveillance, that was a reassortant with H5N1 clade 2.3.4 and H6N6 viruses, that was closely related to the human isolates (Bi et al., 2015a)

**References:**

Abolnik, C., 2007. Molecular Characterization of H5N2 Avian Influenza Viruses Isolated from South African Ostriches in 2006. Avian Dis. 51, 873–879.

Bi, Y., Mei, K., Shi, W., Liu, D., Yu, X., Gao, Z., Zhao, L., Gao, G.F., Chen, J., Chen, Q., 2015a. Two novel reassortants of avian influenza A (H5N6) virus in China. J. Gen. Virol. 96, 975–981. doi:10.1099/vir.0.000056

Bi, Y., Zhang, Z., Liu, W., Yin, Y., Hong, J., Li, X., Wang, H., Wong, G., Chen, J., Li, Y., Ru, W., Gao, R., Liu, D., Liu, Y., Zhou, B., Gao, G.F., Shi, W., Lei, F., 2015b. Highly Pathogenic Avian Influenza A(H5N1) Virus Struck Migratory Birds in China in 2015. Sci. Reports 5. doi:10.1038/srep12986

Briand, F.-X., Schmitz, A., Ogor, K., Le Prioux, A., Guillou-Cloarec, C., Guillemoto, C., Allée, C., Le Bras, M.-O., Hirchaud, E., Quenault, H., Touzain, F., Cherbonnel-Pansart, M., Lemaitre, E., Courtillon, C., Gares, H., Daniel, P., Fediaevsky, A., Massin, P., Blanchard, Y., Eterradossi, N., van der Werf, S., Jestin, V., Niqueux, E., 2017. Emerging highly pathogenic H5 avian influenza viruses in France during winter 2015/16: phylogenetic analyses and markers for zoonotic potential. Euro Surveill 22. https://doi.org/10.2807/1560-7917.ES.2017.22.9.30473

Chen, H., Li, Y., Li, Z., Shi, J., Shinya, K., Deng, G., Qi, Q., Tian, G., Fan, S., Zhao, H., Sun, Y., Kawaoka, Y., 2006. Properties and Dissemination of H5N1 Viruses Isolated during an Influenza Outbreak in Migratory Waterfowl in Western China. J. Virol. 80, 5976–5983. doi:10.1128/JVI.00110-06

Claas, E.C., Osterhaus, A.D., van Beek, R., De Jong, J.C., Rimmelzwaan, G.F., Senne, D.A., Krauss, S., Shortridge, K.F., Webster, R.G., 1998. Human influenza A H5N1 virus related to a highly pathogenic avian influenza virus. The Lancet 351, 472–477. doi:10.1016/S0140-6736(97)11212-0

Creanga, A., Thi Nguyen, D., Gerloff, N., Thi Do, H., Balish, A., Dang Nguyen, H., Jang, Y., Thi Dam, V., Thor, S., Jones, J., Simpson, N., Shu, B., Emery, S., Berman, L., Nguyen, H.T., Bryant, J.E., Lindstrom, S., Klimov, A., Donis, R.O., Davis, C.T., Nguyen, T., 2013. Emergence of multiple clade 2.3.2.1 influenza A (H5N1) virus subgroups in Vietnam and detection of novel reassortants. Virology 444, 12–20. doi:10.1016/j.virol.2013.06.005

Duan, L., Bahl, J., Smith, G.J.D., Wang, J., Vijaykrishna, D., Zhang, L.J., Zhang, J.X., Li, K.S., Fan, X.H., Cheung, C.L., Huang, K., Poon, L.L.M., Shortridge, K.F., Webster, R.G., Peiris, J.S.M., Chen, H., Guan, Y., 2008. The development and genetic diversity of H5N1 influenza virus in China, 1996–2006. Virology 380, 243–254. doi:10.1016/j.virol.2008.07.038

García, M., Crawford, J.M., Latimer, J.W., Rivera-Cruz, E., Perdue, M.L., 1996. Heterogeneity in the haemagglutinin gene and emergence of the highly pathogenic phenotype among recent H5N2 avian influenza viruses from Mexico. J. Gen. Virol. 77 ( Pt 7), 1493–1504. doi:10.1099/0022-1317-77-7-1493

Gu, M., Liu, W., Cao, Y., Peng, D., Wang, X., Wan, H., Zhao, G., Xu, Q., Zhang, W., Song, Q., Li, Y., Liu, X., 2011. Novel Reassortant Highly Pathogenic Avian Influenza (H5N5) Viruses in Domestic Ducks, China. Emerg. Infect. Dis. 17, 1060–1063. doi:10.3201/eid1706.101406

Guan, Y., Peiris, J.S.M., Lipatov, A.S., Ellis, T.M., Dyrting, K.C., Krauss, S., Zhang, L.J., Webster, R.G., Shortridge, K.F., 2002a. Emergence of multiple genotypes of H5N1 avian influenza viruses in Hong Kong SAR. Proc. Natl. Acad. Sci. U. S. A. 99, 8950–8955. doi:10.1073/pnas.132268999

Guan, Y., Peiris, M., Kong, K.F., Dyrting, K.C., Ellis, T.M., Sit, T., Zhang, L.J., Shortridge, K.F., 2002b. H5N1 Influenza Viruses Isolated from Geese in Southeastern China: Evidence for Genetic Reassortment and Interspecies Transmission to Ducks. Virology 292, 16–23. doi:10.1006/viro.2001.1207

Guan, Y., Poon, L.L.M., Cheung, C.Y., Ellis, T.M., Lim, W., Lipatov, A.S., Chan, K.H., Sturm-Ramirez, K.M., Cheung, C.L., Leung, Y.H.C., Yuen, K.Y., Webster, R.G., Peiris, J.S.M., 2004. H5N1 influenza: A protean pandemic threat. Proc. Natl. Acad. Sci. U. S. A. 101, 8156–8161. doi:10.1073/pnas.0402443101

Guan, Y., Shortridge, K.F., Krauss, S., Webster, R.G., 1999. Molecular characterization of H9N2 influenza viruses: Were they the donors of the “internal” genes of H5N1 viruses in Hong Kong? Proc. Natl. Acad. Sci. 96, 9363–9367. doi:10.1073/pnas.96.16.9363

Hu, T., Song, J., Zhang, W., Zhao, H., Duan, B., Liu, Q., Zeng, W., Qiu, W., Chen, G., Zhang, Y., Fan, Q., Zhang, F., 2015. Emergence of novel clade 2.3.4 influenza A (H5N1) virus subgroups in Yunnan Province, China. Infect. Genet. Evol. 33, 95–100. doi:10.1016/j.meegid.2015.04.016

Kawaoka, Y., Naeve, C.W., Webster, R.G., 1984. Is virulence of H5N2 influenza viruses in chickens associated with loss of carbohydrate from the hemagglutinin? Virology 139, 303–316.

Kou, Z., Lei, F.M., Yu, J., Fan, Z.J., Yin, Z.H., Jia, C.X., Xiong, K.J., Sun, Y.H., Zhang, X.W., Wu, X.M., Gao, X.B., Li, T.X., 2005. New Genotype of Avian Influenza H5N1 Viruses Isolated from Tree Sparrows in China. J. Virol. 79, 15460–15466. doi:10.1128/JVI.79.24.15460-15466.2005

Ku, K.B., Park, E.H., Yum, J., Kim, J.A., Oh, S.K., Seo, S.H., 2014. Highly Pathogenic Avian Influenza A(H5N8) Virus from Waterfowl, South Korea, 2014. Emerg. Infect. Dis. 20, 1587–1588. doi:10.3201/eid2009.140390

Lam, T.T.-Y., Hon, C.-C., Pybus, O.G., Pond, S.L.K., Wong, R.T.-Y., Yip, C.-W., Zeng, F., Leung, F.C.-C., 2008. Evolutionary and Transmission Dynamics of Reassortant H5N1 Influenza Virus in Indonesia. PLOS Pathog. 4, e1000130. doi:10.1371/journal.ppat.1000130

Lee, C.-C.D., Zhu, H., Huang, P.-Y., Peng, L., Chang, Y.-C., Yip, C.-H., Li, Y.-T., Cheung, C.-L., Compans, R., Yang, C., Smith, D.K., Lam, T.T.-Y., King, C.-C., Guan, Y., 2014. Emergence and Evolution of Avian H5N2 Influenza Viruses in Chickens in Taiwan. J. Virol. 88, 5677–5686. doi:10.1128/JVI.00139-14

Lee, C.-W., Suarez, D.L., Tumpey, T.M., Sung, H.-W., Kwon, Y.-K., Lee, Y.-J., Choi, J.-G., Joh, S.-J., Kim, M.-C., Lee, E.-K., Park, J.-M., Lu, X., Katz, J.M., Spackman, E., Swayne, D.E., Kim, J.-H., 2005. Characterization of Highly Pathogenic H5N1 Avian Influenza A Viruses Isolated from South Korea. J. Virol. 79, 3692–3702. doi:10.1128/JVI.79.6.3692-3702.2005

Lee, Y.-J., Kang, H.-M., Lee, E.-K., Song, B.-M., Jeong, J., Kwon, Y.-K., Kim, H.-R., Lee, K.-J., Hong, M.-S., Jang, I., Choi, K.-S., Kim, J.-Y., Lee, H.-J., Kang, M.-S., Jeong, O.-M., Baek, J.-H., Joo, Y.-S., Park, Y.H., Lee, H.-S., 2014. Novel Reassortant Influenza A(H5N8) Viruses, South Korea, 2014. Emerg. Infect. Dis. 20, 1087–1089. doi:10.3201/eid2006.140233

Li, Y., Shi, J., Zhong, G., Deng, G., Tian, G., Ge, J., Zeng, X., Song, J., Zhao, D., Liu, L., Jiang, Y., Guan, Y., Bu, Z., Chen, H., 2010. Continued Evolution of H5N1 Influenza Viruses in Wild Birds, Domestic Poultry, and Humans in China from 2004 to 2009. J. Virol. 84, 8389–8397. doi:10.1128/JVI.00413-10

Liu, C.-G., Liu, M., Liu, F., Lv, R., Liu, D.-F., Qu, L.-D., Zhang, Y., 2013. Emerging multiple reassortant H5N5 avian influenza viruses in ducks, China, 2008. Vet. Microbiol. 167, 296–306. doi:10.1016/j.vetmic.2013.09.004

Lu, L., Lycett, S.J., Leigh Brown, A.J., 2014. Determining the Phylogenetic and Phylogeographic Origin of Highly Pathogenic Avian Influenza (H7N3) in Mexico. PLoS ONE 9, e107330. doi:10.1371/journal.pone.0107330

Mi, Z., Liu, W., Fan, H., An, X., Pei, G., Wang, W., Xu, X., Ma, M., Zhang, Z., Cao, W., Tong, Y., 2013. Complete Genome Sequence of Avian Influenza Virus A/chicken/Jiangsu/1001/2013(H5N2), Demonstrating Continuous Reassortance of H5N2 in China. Genome Announc. 1, e00469–13. doi:10.1128/genomeA.00469-13

Monne, I., Joannis, T.M., Fusaro, A., De Benedictis, P., Lombin, L.H., Ularamu, H., Egbuji, A., Solomon, P., Obi, T.U., Cattoli, G., Capua, I., 2008. Reassortant Avian Influenza Virus (H5N1) in Poultry, Nigeria, 2007. Emerg. Infect. Dis. 14, 637–640. doi:10.3201/eid1404.071178

Monne, I., Meseko, C., Joannis, T., Shittu, I., Ahmed, M., Tassoni, L., Fusaro, A., Cattoli, G., 2015. Highly Pathogenic Avian Influenza A(H5N1) Virus in Poultry, Nigeria, 2015. Emerg. Infect. Dis. 21, 1275–1277. doi:10.3201/eid2107.150421

Monne, I., Yamage, M., Dauphin, G., Claes, F., Ahmed, G., Giasuddin, M., Salviato, A., Ormelli, S., Bonfante, F., Schivo, A., Cattoli, G., 2013. Reassortant Avian Influenza A(H5N1) Viruses with H9N2-PB1 Gene in Poultry, Bangladesh. Emerg. Infect. Dis. 19, 1630–1634. doi:10.3201/eid1910.130534

Nguyen, T., Rivailler, P., Davis, C.T., Thi Hoa, D., Balish, A., Hoang Dang, N., Jones, J., Thi Vui, D., Simpson, N., Thu Huong, N., Shu, B., Loughlin, R., Ferdinand, K., Lindstrom, S.E., York, I.A., Klimov, A., Donis, R.O., 2012. Evolution of highly pathogenic avian influenza (H5N1) virus populations in Vietnam between 2007 and 2010. Virology 432, 405–416. doi:10.1016/j.virol.2012.06.021

Owoade, A.A., Gerloff, N.A., Ducatez, M.F., Taiwo, J.O., Kremer, J.R., Muller, C.P., 2008. Replacement of Sublineages of Avian Influenza (H5N1) by Reassortments, Sub-Saharan Africa. Emerg. Infect. Dis. 14, 1731–1735. doi:10.3201/eid1411.080555

Pan, M., Gao, R., Lv, Q., Huang, S., Zhou, Z., Yang, L., Li, X., Zhao, X., Zou, X., Tong, W., Mao, S., Zou, S., Bo, H., Zhu, X., Liu, L., Yuan, H., Zhang, M., Wang, Daqing, Li, Z., Zhao, W., Ma, M., Li, Y., Li, T., Yang, H., Xu, J., Zhou, L., Zhou, X., Tang, W., Song, Y., Chen, T., Bai, T., Zhou, J., Wang, Dayan, Wu, G., Li, D., Feng, Z., Gao, G.F., Wang, Y., He, S., Shu, Y., 2016. Human infection with a novel, highly pathogenic avian influenza A (H5N6) virus: Virological and clinical findings. J. Infect. 72, 52–59. doi:10.1016/j.jinf.2015.06.009

Pasick, J., Berhane, Y., Joseph, T., Bowes, V., Hisanaga, T., Handel, K., Alexandersen, S., 2015. Reassortant Highly Pathogenic Influenza A H5N2 Virus Containing Gene Segments Related to Eurasian H5N8 in British Columbia, Canada, 2014. Sci. Reports 5. doi:10.1038/srep09484

Salzberg, S.L., Kingsford, C., Cattoli, G., Spiro, D.J., Janies, D.A., Aly, M.M., Brown, I.H., Couacy-Hymann, E., De Mia, G.M., Dung, D.H., Guercio, A., Joannis, T., Ali, A.S.M., Osmani, A., Padalino, I., Saad, M.D., Savić, V., Sengamalay, N.A., Yingst, S., Zaborsky, J., Zorman-Rojs, O., Ghedin, E., Capua, I., 2007. Genome Analysis Linking Recent European and African Influenza (H5N1) Viruses. Emerg. Infect. Dis. 13, 713–718. doi:10.3201/eid1305.070013

Shi, W.F., Gibbs, M.J., Zhang, Y.Z., Zhang, Z., Zhao, X.M., Jin, X., Zhu, C.D., Yang, M.F., Yang, N.N., Cui, Y.J., Ji, L., 2008. Genetic analysis of four porcine avian influenza viruses isolated from Shandong, China. Arch. Virol. 153, 211–217. doi:10.1007/s00705-007-1083-1

Smith, G.J.D., Fan, X.H., Wang, J., Li, K.S., Qin, K., Zhang, J.X., Vijaykrishna, D., Cheung, C.L., Huang, K., Rayner, J.M., Peiris, J.S.M., Chen, H., Webster, R.G., Guan, Y., 2006. Emergence and predominance of an H5N1 influenza variant in China. Proc. Natl. Acad. Sci. 103, 16936–16941. doi:10.1073/pnas.0608157103

Sturm-Ramirez, K.M., Ellis, T., Bousfield, B., Bissett, L., Dyrting, K., Rehg, J.E., Poon, L., Guan, Y., Peiris, M., Webster, R.G., 2004. Reemerging H5N1 Influenza Viruses in Hong Kong in 2002 Are Highly Pathogenic to Ducks. J. Virol. 78, 4892–4901. doi:10.1128/JVI.78.9.4892-4901.2004

Subbarao, K., Klimov, A., Katz, J., Regnery, H., Lim, W., Hall, H., Perdue, M., Swayne, D., Bender, C., Huang, J., Hemphill, M., Rowe, T., Shaw, M., Xu, X., Fukuda, K., Cox, N., 1998. Characterization of an Avian Influenza A (H5N1) Virus Isolated from a Child with a Fatal Respiratory Illness. Science 279, 393–396. doi:10.1126/science.279.5349.393

Tabynov, K., Sansyzbay, A., Sandybayev, N., Mambetaliyev, M., 2014. The pathogenicity of swan derived H5N1 virus in birds and mammals and its gene analysis. Virol. J. 11, 207. doi:10.1186/s12985-014-0207-y

Thor, S.W., Nguyen, H., Balish, A., Hoang, A.N., Gustin, K.M., Nhung, P.T., Jones, J., Thu, N.N., Davis, W., Ngoc, T.N.T., Jang, Y., Sleeman, K., Villanueva, J., Kile, J., Gubareva, L.V., Lindstrom, S., Tumpey, T.M., Davis, C.T., Long, N.T., 2015. Detection and Characterization of Clade 1 Reassortant H5N1 Viruses Isolated from Human Cases in Vietnam during 2013. PLoS ONE 10, e0133867. doi:10.1371/journal.pone.0133867

Torchetti, M.K., Killian, M.L., Dusek, R.J., Pedersen, J.C., Hines, N., Bodenstein, B., White, C.L., Ip, H.S., 2015. Novel H5 Clade 2.3.4.4 Reassortant (H5N1) Virus from a Green-Winged Teal in Washington, USA. Genome Announc. 3. doi:10.1128/genomeA.00195-15

Wan, X.-F., Nguyen, T., Davis, C.T., Smith, C.B., Zhao, Z.-M., Carrel, M., Inui, K., Do, H.T., Mai, D.T., Jadhao, S., Balish, A., Shu, B., Luo, F., Emch, M., Matsuoka, Y., Lindstrom, S.E., Cox, N.J., Nguyen, C.V., Klimov, A., Donis, R.O., 2008. Evolution of Highly Pathogenic H5N1 Avian Influenza Viruses in Vietnam between 2001 and 2007. PLoS ONE 3. doi:10.1371/journal.pone.0003462

Wong, F.Y.K., Phommachanh, P., Kalpravidh, W., Chanthavisouk, C., Gilbert, J., Bingham, J., Davies, K.R., Cooke, J., Eagles, D., Phiphakhavong, S., Shan, S., Stevens, V., Williams, D.T., Bounma, P., Khambounheuang, B., Morrissy, C., Douangngeun, B., Morzaria, S., 2015. Reassortant Highly Pathogenic Influenza A(H5N6) Virus in Laos. Emerg. Infect. Dis. 21, 511–516. doi:10.3201/eid2103.141488

Wu, H., Peng, X., Xu, L., Jin, C., Cheng, L., Lu, X., Xie, T., Yao, H., Wu, N., 2014. Novel Reassortant Influenza A(H5N8) Viruses in Domestic Ducks, Eastern China. Emerg. Infect. Dis. 20, 1315–1318. doi:10.3201/eid2008.140339

Xu, X., Subbarao, K., Cox, N.J., Guo, Y., 1999. Genetic Characterization of the Pathogenic Influenza A/Goose/Guangdong/1/96 (H5N1) Virus: Similarity of Its Hemagglutinin Gene to Those of H5N1 Viruses from the 1997 Outbreaks in Hong Kong. Virology 261, 15–19. doi:10.1006/viro.1999.9820

Yu, Y., Wang, X., Jin, T., Wang, H., Si, W., Yang, H., Wu, J., Yan, Y., Liu, G., Sang, X., Gao, Y., Xia, X., Yu, X., Pan, J., Gao, G.F., Zhou, J., 2015. Newly-emergent highly pathogenic H5N9 subtype avian influenza A virus. J. Virol. JVI.00653–15. doi:10.1128/JVI.00653-15

Zhang, R., Chen, T., Ou, X., Liu, R., Yang, Y., Ye, W., Chen, J., Yao, D., Sun, B., Zhang, X., Zhou, J., Sun, Y., Chen, F., Wang, S.-P., 2016. Clinical, epidemiological and virological characteristics of the first detected human case of avian influenza A(H5N6) virus. Infect. Genet. Evol. 40, 236–242. doi:10.1016/j.meegid.2016.03.010

Zhao, G., Gu, X., Lu, X., Pan, J., Duan, Z., Zhao, K., Gu, M., Liu, Q., He, L., Chen, J., Ge, S., Wang, Y., Chen, S., Wang, X., Peng, D., Wan, H., Liu, X., 2012. Novel Reassortant Highly Pathogenic H5N2 Avian Influenza Viruses in Poultry in China. PLoS ONE 7, e46183. doi:10.1371/journal.pone.0046183

Zhao, K., Gu, M., Zhong, L., Duan, Z., Zhang, Y., Zhu, Y., Zhao, G., Zhao, M., Chen, Z., Hu, S., Liu, W., Liu, Xiaowen, Peng, D., Liu, Xiufan, 2013. Characterization of three H5N5 and one H5N8 highly pathogenic avian influenza viruses in China. Vet. Microbiol. 163, 351–357. doi:10.1016/j.vetmic.2012.12.025
