# Supplementary material for: Geographical and Historical Patterns in the Emergences of Novel Highly Pathogenic Avian Influenza (HPAI) H5 and H7 Viruses in Poultry
Source: Front Vet Sci. 2018 Jun 5;5:84. doi: 10.3389/fvets.2018.00084 (PMC5996087; doi:10.3389/fvets.2018.00084)
Supplement: Supplementary file 4 [file Table2.DOCX]

Table-2. Numbers of sequences of Influenza virus A for Avian hosts submitted in the National Center for Biotechnology Information (NCBI) Influenza Virus Database (https://www.ncbi.nlm.nih.gov/genomes/FLU/Database/nph-select.cgi?go=database) during three time periods (1960-1995, 1996-2005, and 2006-2015) for a subset of countries (Egypt, Viet Nam, Hong-Kong, Bangladesh, United States of America, China, Indonesia). The number of chicken (x10^3^, FAOSTAT) per country for three years (1978, 2000 and 2010) was also mentioned in the table. The total number of sequences submitted and the total number of chicken heads (10^3^) recorded in the database over the study period was mentioned in the table as well as the contribution of each country in the total of each column (%).

| **Country** | **Number of sequences**  **(1960-1995)** | **Chicken stock (1978)** | **Number of sequences**  **(1996-2005)** | **Chicken stock (2000)** | **Number of sequences**  **(2006-2015)** | **Chicken stock (2010)** |
| --- | --- | --- | --- | --- | --- | --- |
| Egypt | 0 (0.00%) | 26986 (0.48%) | 55 (0.12%) | 89000 (0.62%) | 3637 (2.27%) | 117500 (0.58%) |
| Viet Nam | 0 (0.00%) | 39800 (0.71%) | 1728 (3.87%) | 137300 (0.95%) | 7688 (4.81%) | 218201 (1.08%) |
| Hong Kong | 566 (3.07%) | - | 1912 (4.28%) | - | 2019 (1.26%) | - |
| Bangladesh | 0 (0.00%) | 66817 (1.20%) | 0 (0.00%) | 132700 (0.92%) | 2095 (1.31%) | 228035 (1.13%) |
| USA | 11555 (62.60%) | 1001000 (17.91%) | 12492 (27.98%) | 1860000 (12.89%) | 58298 (36.45%) | 1956000 (9.69%) |
| China | 106 (0.57%) | 820365 (14.67%) | 12567 (28.15%) | 3623012 (25.11%) | 39396 (24.63%) | 5302720 (26.28%) |
| Indonesia | 0 (0.00%) | 114987 (2.06%) | 713 (1.60%) | 859497 (5.96%) | 859 (0.54%) | 1349626 (6.69%) |
| Total | 18457 | 5590416 | 44639 | 14430645 | 159934 | 20177368 |

| Country | nbSqTi1 | FAO78 | nbSqTi2 | FAO00 | nbSqTi3 | FAO10 |
| --- | --- | --- | --- | --- | --- | --- |
| Egypt | 0 (0.00%) | 26986 (0.48%) | 55 (0.12%) | 89000 (0.62%) | 3637 (2.27%) | 117500 (0.58%) |
| Viet Nam | 0 (0.00%) | 39800 (0.71%) | 1728 (3.87%) | 137300 (0.95%) | 7688 (4.81%) | 218201 (1.08%) |
| Hong Kong | 566 (3.07%) | NA ( NA%) | 1912 (4.28%) | NA ( NA%) | 2019 (1.26%) | NA ( NA%) |
| Bangladesh | 0 (0.00%) | 66817 (1.20%) | 0 (0.00%) | 132700 (0.92%) | 2095 (1.31%) | 228035 (1.13%) |
| USA | 11555 (62.60%) | 1001000 (17.91%) | 12492 (27.98%) | 1860000 (12.89%) | 58298 (36.45%) | 1956000 (9.69%) |
| China | 106 (0.57%) | 820365 (14.67%) | 12567 (28.15%) | 3623012 (25.11%) | 39396 (24.63%) | 5302720 (26.28%) |
| Indonesia | 0 (0.00%) | 114987 (2.06%) | 713 (1.60%) | 859497 (5.96%) | 859 (0.54%) | 1349626 (6.69%) |
| Total | 18457 | 5590416 | 44639 | 14430645 | 159934 | 20177368 |

the National Institute of Health which assembled the larger genetic sequence database through the National Center for Biotechnology Information (NCBI) portal and the GenBank and the Influenza Genome Sequencing Project.

To illustrate the recent trends in AI diseases surveillance and monitoring, all the nucleic acid sequence records up to the 31 December 2017 were extracted from the NCBI Influenza Virus Database (// The Influenza Virus Resource at the National Center for Biotechnology Information).
